# Proteomic Insights into the Immune and Sex-Specific Proteins in the Skin Mucus of Barramundi (*Lates calcarifer*)

**DOI:** 10.3390/proteomes14010015

**Published:** 2026-03-20

**Authors:** Varsha V. Balu, Dean R. Jerry, Andreas L. Lopata

**Affiliations:** 1 ARC Industrial Transformation Research Hub for Supercharging Tropical Aquaculture Through Genetic Solutions, James Cook University, Townsville, QLD 4811, Australia; 2 Centre for Sustainable Tropical Fisheries and Aquaculture, College of Science and Engineering, James Cook University, Townsville, QLD 4811, Australia; 3 Molecular Allergy Research Laboratory, College of Science and Engineering, James Cook University, Townsville, QLD 4811, Australia; 4 Tropical Futures Institute, James Cook University, Singapore 387380, Singapore

**Keywords:** Asian seabass, gene ontology, KEGG pathway, COG functional classification, mucus proteins, aquaculture

## Abstract

Background: Fish skin mucus contains proteins involved in diverse biological pathways, representing a valuable non-invasive diagnostic of fish health. Methods: Skin mucus from three male and three female barramundi was analysed using liquid chromatography-tandem mass spectrometry (LC-MS/MS) following protein extraction and S-Trap digestion. Results and Discussion: A total of 1801 protein groups were matched to the *L. calcarifer* reference proteome and functionally annotated using Gene Ontology (GO) terms via UniProt ID mapping, with representation across Biological Process, Cellular Component, and Molecular Function categories. Functional classification using eggNOG-mapper further associated leading protein group sequences with Clusters of Orthologous Groups (COGs) and Kyoto Encyclopaedia of Genes and Genomes (KEGG) pathways. GO-based screening prioritised 352 putatively immune-relevant protein groups and 24 protein groups associated with sex- and reproduction-related processes, highlighting the functional complexity of the skin mucus proteome. Comparative analysis revealed sex-associated patterns in protein group detection and relative abundance, with differential abundance analysis identifying 244 protein groups exhibiting statistically significant differences between male and female samples. Conclusions: This study provides the first comprehensive discovery-based characterisation of the barramundi skin mucus proteome and establishes a baseline reference dataset for this aquaculture-relevant species. The findings support the utility of skin mucus proteomics for exploring immune and sex-associated molecular patterns and provide a baseline dataset for future validation studies investigating non-invasive health and reproductive monitoring.

## 1. Introduction

Barramundi is a commercially important aquaculture and fisheries species native to Australia, Papua New Guinea, and Southeast Asia. Its high nutritional value, strong market demand, unique physiological traits (i.e., euryhalinity, protandrous hermaphrodite), and relative ease of cultivation contribute to its widespread popularity and economic importance [1,2]. This species is now intensively farmed on four continents [3,4], and in Australia, the barramundi industry is projected to be valued at AUD 200 M by 2030 [5].

Barramundi are protandrous sequential hermaphrodites, starting life as males, maturing at 2–4 years, and typically change into females between 4 and 8 years of age or when they reach ~60 cm [6,7,8]. Consequently, sex in barramundi is closely linked to age, size and reproductive maturation stages, reflecting a plastic form of sex determination influenced by developmental and environmental factors [8]. This means comparison between males and females may be confounded, as physiological or molecular differences may partly reflect differences in maturation stage or the process of sex transition rather than inherent sex-specific traits. Fish health monitoring is an essential component of aquaculture and is often conducted through blood collection, which is invasive and can sometimes be detrimental to the health of the fish [6,9]. Protandry adds an additional complication in the barramundi breeding process, with sex determination and maturation status a particular challenge in broodstock management [10]. Due to no physical differences evident between males and females, sexual and gonadal maturation status are presently determined through invasive cannulation with a plastic tube and aspirating reproductive tissue to assess if sperm or oocytes are present [11] or through blood analysis [12]. Because broodstock are valuable and this process is stressful to broodstock, the ability to monitor health and reproductive status non-invasively is particularly important for barramundi aquaculture.

Fish are constantly in contact with their aquatic environment, with their skin mucus acting as the first barrier between the organism and its dynamic surroundings [13]. Fish skin mucus, much like other mucosal membranes, plays a vital role in the health and well-being of the fish. This mucus is highly adaptive and is known to play diverse roles in both specific and general biological processes of fish, including osmoregulation, disease resistance, respiration, nutrition, and communication [14,15,16,17]. Fish skin mucus is composed of different proteins, lipids, carbohydrates, DNA and RNA, along with multiple protein-cell networks, protein-protein networks and immune defence and stress molecules [18]. It is also known to contain hormones derived from epithelial cells, immune cells, and the circulatory system [19], allowing it to reflect the physiological state of the host [20]. In teleosts, hormones and endocrine signals can diffuse from the bloodstream into mucus through epithelial tissues, meaning that mucus composition may respond to endocrine regulation associated with stress, immune activation, and reproduction [14,21]. As reproductive maturation and sex transition in barramundi are driven by substantial endocrine changes, including shifts in steroid hormone profiles, these systemic signals may influence the molecular composition of skin mucus. Consequently, mucus proteomes may provide insight into physiological processes linked to endocrine status and reproductive stage. Overall, skin mucus is known to be a good representation of the health status of aquatic animals and allows for minimally invasive monitoring. However, despite barramundi being widely studied, little work has examined the potential for such non-invasive sampling approaches, and the species’ proteome to date remains largely uncharacterised.

With advancements in proteomic tools and increasing focus on non-invasive diagnostics, studies have explored various elements of fish skin mucosa. Several studies have characterised the skin mucus proteome to establish baselines in commercially valuable species such as European sea bass (*Dicentrarchus labrax*) [22], gilthead sea bream (*Sparus aurata*) [23] and Atlantic salmon (*Salmo salar*) [24]. Other proteomic studies on fish skin mucus have been used to investigate differential expression or abundance of proteins to screen for disease in aquaculture, such as fat greenling (*Hexagrammos otakii*) infected with *Vibrio harveyi* [25], crucian carp (*Carassius carassius*) infected with *Gyrodactylus kobayashii* [26], Rohu (*Labeo rohita*) challenged with *Aeromonas hydrophila* [27], turbot challenged with *Vibrio anguillarum* [28] and greater amberjack (*Seriola dumerili*) infected with *Neobenedenia girellae* [13]. Studies also have identified biomarkers indicative of stress in the fish (e.g., in gilthead sea bream (*Sparus aurata*) [21,29]), as well as the antimicrobial potential of epidermal mucus from marine fish [30]. More recently, studies have been conducted on gender differences, as in the case of discus fish (*Symphysodon haraldi*) and sex-specific immune markers from the skin mucus of Rainbow trout (*Oncorhynchus mykiss*) have been investigated in skin mucus [31,32]. Therefore, integrating proteomic tools into aquaculture diagnostics can aid in the development of less invasive approaches for detecting stress, disease, or reproductive status in broodstock fish. More broadly, recent studies across aquaculture have explored advanced phenotyping and data-driven technologies to improve monitoring and breeding strategies, highlighting the growing role of novel biological and technological tools in modern aquaculture systems [33]. For such approaches to be effective, however, baseline molecular datasets are required. Establishing reference skin mucus proteomes for species of interest, therefore, represents an important first step.

In this study, LC-MS/MS was employed to characterise the protein composition of barramundi skin mucus and to generate the first discovery-based skin mucus proteome dataset for this species. Protein groups inferred from database-matched peptide evidence were functionally annotated using GO, COG, and KEGG classifications. These annotations were subsequently screened to prioritise putatively immune-related and sex-associated protein groups. Throughout this study, the term ‘proteoforms’ is used in a descriptive and inferential context to refer to protein group-level molecular variants suggested by peptide-level mass spectrometry data. These inferred proteoforms represent database-matched protein entries and do not constitute direct analytical resolution of discrete post-translationally modified or sequence-distinct protein species.

## 2. Materials and Methods

### 2.1. Sample Collection

Broodstock were maintained in a recirculating aquaculture system operating under ambient environmental temperatures, with salinity maintained at 30 ppt. Fish were fed a commercial broodstock diet once daily. Three male and three female mature broodstock barramundi, with individuals ~85 cm in length, were sampled. Fish sex was identified based on historical records and confirmed via cannulation (Animal ethics approval: A2962, James Cook University); however, detailed gonadal staging was not available, and reproductive maturity may therefore have influenced the presence or absence of putative reproductive protein groups in mucus. Fish were individually sedated in a tub of AQUI-S (AQUI-S New Zealand Ltd., Lower Hutt, New Zealand) (40 ppm) for 2–3 min until rapid sedation was achieved. Fish were placed on a clean tarp, and skin mucus was collected using a cotton swab by firmly rubbing along the lateral surface cephalocaudally and avoiding the ventral side. Each fish was turned and sampled on the other side with a new cotton swab. Swabs were immediately placed into pre-labelled tubes, stored on ice during collection, and subsequently transferred to −80 °C for storage until further processing to minimise proteolytic degradation. While blank swab controls were not included in this study, sampling was undertaken to minimise the introduction of background proteins and contaminants.

### 2.2. Protein Extraction and Digestion

Frozen skin mucus samples were thawed on ice and 500 μL of phosphate-buffered saline (PBS, pH 7.4), prepared in-house according to standard laboratory protocols, was added to each tube (per mucus swab). Samples were incubated on a shaker incubator at 60 rpm overnight (~16 h) at 4 °C to solubilise proteins. Following incubation, both the liquid fraction and swabs were transferred to Costar Spin-X centrifuge tubes fitted with 0.22 µm cellulose acetate filters (Corning Inc., Coring, NY, USA) and centrifuged at 5000× *g* for 10 min at 4 °C. This step was repeated until all sample material had passed through the filter, with a maximum of three centrifugation cycles. For each biological sample, protein extracts from both sides of the fish were combined to minimise side-specific bias as previously reported in Senegalese sole mucus proteomics studies [34]. Protein concentration was determined using the bicinchoninic acid (BCA) assay with the BCA Protein Assay Kit (Pierce, Thermo Fisher Scientific, Waltham, MA, USA) and Bovine Serum Albumin (BSA) Standard Pre-Diluted Set (Pierce, Thermo Fisher Scientific, Waltham, MA, USA). Sample volumes were normalised to 100 µg of protein, and precipitated with four times the volume of ice-cold HPLC-grade acetone (Merck Life Science Pty Ltd., Bayswater, VIC, Australia). Protein pellets were dried in a Speed-Vac and resuspended in 100 μL of lysis buffer (10% sodium dodecyl sulphate (SDS), 100 mM triethylammonium bicarbonate (TEAB) pH 8.5, 8 M Urea; all reagents from Sigma-Aldrich, St. Louis, MO, USA). To assess protein extraction quality and visualise the overall protein composition of barramundi skin mucus samples prior to LC-MS/MS analysis, sodium dodecyl sulfate polyacrylamide gel electrophoresis (SDS-PAGE) was performed as previously described [35,36]. Mucus protein extracts were separated on 12% polyacrylamide gels under denaturing conditions and stained with Coomassie Brilliant Blue (Bio-Rad Laboratories, Hercules, CA, USA) to visualise protein bands. The resulting banding patterns were used as a qualitative assessment of protein integrity and sample complexity. A representative gel image is provided in the Supplementary Material (Figure S1).

### 2.3. LC-MS/MS Sample Prep and Processing

For mass spectrometric analysis, 23 μL of sample (~23 μg of protein) was subjected to in-solution digest using the Protifi S-trap Micro protocol [37] and as previously described [38] with some modifications. Samples were reduced with 0.5 μL of 10 mM Tris (2-carboxyethyl) phosphine hydrochloride (TCEP; Sigma-Aldrich, St. Louis, MO, USA), incubated at 55 °C for 15 min, alkylated with 2.5 μL of 50 mM iodoacetamide (IAA; Sigma-Aldrich, St. Louis, MO, USA) and subsequently incubated in the dark at room temperature for 45 min. Proteins were acidified using 2.5 μL of 2.5% phosphoric acid (Thermo Fisher Scientific, Waltham, MA, USA) and vortexed. Then, the proteins were washed using 165 μL of a binding/wash buffer made with 100 mM TEAB in 90% high performance liquid chromatography (HPLC) grade methanol (Thermo Fisher Scientific, Waltham, MA, USA). Samples were loaded into a Protifi S-Trap Micro columns (Protifi LLC, Huntington, NY, USA), placed in a flow-through collection tube, and only 80 μL was added at a time. The S-Trap and collection tubes were centrifuged at 4000× *g* for 30 s to trap proteins, and this process was repeated until all the sample had passed through the S-Trap. To each S-Trap column, 150 μL of binding/wash buffer was added and centrifuged at 4000× *g* for 30 s and repeated three times. S-Traps were centrifuged once more at 4000× *g* for 1 min to fully remove binding/wash buffer, and the columns were moved to clean 1.5 mL Eppendorf tubes. Proteins were digested with 50 μL of digestion buffer containing 1 μg of Mass Spectrometry-grade Porcine Trypsin (Sigma-Aldrich, St. Louis, MO, USA), 1 mM hydrochloric acid (HCl; Thermo Fisher Scientific, Waltham, MA, USA) and 50 mM TEAB, incubated overnight at 37 °C. Peptides were sequentially eluted using 40 μL each of 50 mM TEAB, 0.2% formic acid (Sigma- Aldrich, St. Louis, MO, USA), and 50% acetonitrile (Thermo Fisher Scientific, Waltham, MA, USA). Organic solvent was partially removed by vacuum centrifugation, and samples were freeze-dried prior to shipment to the Bio21 Molecular Science and Biotechnology Institute (University of Melbourne) for LC-MS/MS analysis.

Dried peptide samples were reconstituted in 3% acetonitrile with 0.1% formic acid and peptides were separated by nano-flow reversed-phase liquid chromatography and introduced into an Orbitrap Exploris 480 mass spectrometer (Thermo Fisher Scientific, Waltham, MA, USA) via nano-electrospray ionisation (NSI) operating in positive ion mode. Mass spectrometric data were acquired using data-dependent acquisition (DDA), employing a facility-standard discovery proteomics method. Full-scan MS spectra were acquired in the Orbitrap analyser across a precursor mass range of 375–1500 *m*/*z*, consistent with typical peptide detection, followed by sequential selection of multiply charged precursor ions for tandem mass spectrometry (MS/MS). Fragmentation was performed using higher-energy collisional dissociation (HCD), and MS/MS spectra were recorded in the Orbitrap analyser. Instrument control and data acquisition were performed using Thermo Scientific Xcalibur software (version 4.1; Thermo Fisher Scientific, Waltham, MA, USA). Raw data files (.raw) were generated for each individual sample injection. Each biological sample was analysed using a single LC-MS/MS injection, with no technical replication due to sample availability and cost of sample processing. The analytical workflow, therefore, prioritised biological sampling across individuals rather than repeated technical measurements. As a result, the dataset should be interpreted primarily as a discovery-based survey of the mucus proteome rather than a fully quantitative assessment of protein abundance.

### 2.4. Database Searching and Protein Group Assignment

Raw data from mass spectrometry of mucus samples from the six specimens were analysed using MaxQuant (Version 2.6.7.0) [39] with the integrated Andromeda search engine. Database searching was performed against a UniProt reference proteome for *Lates calcarifer* (organism ID: 8187), including both canonical protein sequences and annotated isoforms. Samples were analysed as single, unfractionated LC-MS/MS runs, with experiments defining biological replicates by sex (male and female). Trypsin/P was specified as the digestion enzyme with up to two missed cleavages permitted. Carbamidomethylation of cysteine residues was specified as a fixed modification, and oxidation (M) and deamidation (NQ) were included as variable modifications. Peptide and protein identifications were filtered using a 1% false discovery rate (FDR) determined by a target-decoy approach. The minimum peptide length was set to seven amino acids, and the minimum number of peptides required for protein identification was one unique peptide. Label-free quantification (LFQ) was enabled using default MaxQuant parameters to allow relative abundance comparisons across samples. Protein inference was conducted by MaxQuant using the protein group approach, whereby peptide evidence may map to one or more protein entries. Importantly, proteins reported in this study represent database-matched protein groups inferred from peptide evidence, rather than analytically confirming individual proteoforms.

### 2.5. Data Analysis and Statistics

The proteinGroups.txt file generated by MaxQuant was imported into Perseus (Version 2.1.3.0) [40]. The data was filtered and rows that were ‘Only identified by site’, contained ‘Reverse’ reads and also contained ‘Potential contaminants’ values were removed. The LFQ intensity data for each sample were then log_2_-transformed to create a normal data distribution. Histograms were created to check for normal distribution, and summary statistics were generated to undertake quality control for the data. The log transformed LFQ intensities file generated from Perseus was exported as a .txt file loaded into R (Version 4. 4. 2) [41] and R Studio (Version 2024.12.1.563) [42] for all downstream data analysis.

#### 2.5.1. Proteome Annotation and Gene Ontology Mapping

For functional annotation, UniProt accession numbers corresponding to the leading protein (the highest number of peptides identified from a protein in a group [43]) were extracted from the ‘Majority protein IDs’ column in the proteinGroups.txt output file from MaxQuant. These accession numbers were mapped using the UniProt ID mapping platform and GO annotations across Biological Process, Molecular Function, and Cellular Component categories were retrieved and analysed in R Studio.

#### 2.5.2. COG Functional Classification and KEGG Pathway Mapping

FASTA sequences corresponding to the leading proteins from each protein group were downloaded as zipped FASTA files (.gz) from UniProt ID mapper and uploaded to eggNOG-mapper v2 [44]. Using the eggNOG 5.0 database [45] as a reference, proteins were assigned to COG functional categories and KEGG Orthology pathways based on sequence homology. The resulting output file was loaded into R Studio for further analysis.

#### 2.5.3. Immune-Relevant Protein Group Screening

Custom R functions were developed to screen protein groups for immune-relevant functional annotations based on curated GO anchor terms. Biological Process GO terms were filtered using immune-associated terms under the umbrella of ‘immune system process [GO:0002376]’, ‘immune response [GO:0006955]’, ‘innate immune response [GO:0045087]’, ‘adaptive immune response [GO:0002250]’, and ‘regulation of immune system process [GO:0002682]’. GO terms under Molecular Function annotations were screened using the immune specific anchor terms such as ‘antigen binding [GO:0003823]’, ‘pattern recognition receptor activity [GO:0038187]’, ‘cytokine activity [GO:0005125]’, ‘chemokine activity [GO:0008009]’, ‘complement receptor activity [GO:0004875]’, ‘lysozyme activity [GO:0003796]’, ‘chitinase activity [GO:0004568]’, ‘lipopolysaccharide binding [GO:0001530]’, ‘peptidoglycan binding [GO:0042834]’, ‘scavenger receptor activity [GO:0005044]’, ‘N-acetylmuramoyl-L-alanine amidase activity [GO:0008745]’, ‘peroxidase activity [GO:0004601]’, and ‘peptidase inhibitor activity [GO:0030414]’. To cover immune-relevant proteins from the Cellular Component, GO terms were filtered using immune compartment terms such as ‘MHC class I protein complex [GO:0042612]’, ‘MHC class II protein complex [GO:0042613]’, ‘phagocytic vesicle [GO:0045335]’, ‘membrane attack complex [GO:0005579]’, ‘autophagosome [GO:0005776]’, ‘fibrinogen complex [GO:0005577]’, ‘immunoglobulin complex [GO:0019814]’, ‘immunological synapse [GO:0001772]’, ‘secretory granule [GO:0030141]’, ‘extracellular vesicle [GO:1903561]’, ‘extracellular region [GO:0005576]’ and ‘extracellular exosome [GO:0070062]’. These screening steps were used to prioritise putatively immune-relevant protein groups rather than to assert definitive immune function.

#### 2.5.4. Sex-Specific and Reproductive Protein Screening and Differentially Abundant Protein (DAP) Analyses

Protein groups were similarly screened for reproduction-relevant and sex-specific annotations. Biological Process GO terms were screened using anchor terms—‘reproductive process [GO:0022414]’, ‘sexual reproduction [GO:0019953]’, ‘gamete generation [GO:0007276]’, ‘spermatogenesis [GO:0007283]’, ‘oogenesis [GO:0048477]’, ‘sex differentiation [GO:0007548]’, ‘gonad development [GO:0008406]’, ‘reproductive system development [GO:0061458]’, ‘hormone metabolic process [GO:0042445]’, ‘steroid biosynthetic process [GO:0006694]’, ‘fertilization [GO:0009566]’, ‘single fertilization [GO:0007338]’, ‘sperm egg recognition [GO:0035036]’, ‘binding of sperm to zona pellucida [GO:0007339]’, ‘acrosome reaction [GO:0007340]’, ‘oocyte maturation [GO:0001556]’, ‘ovulation [GO:0030728]’, ‘meiotic cell cycle [GO:0051321]’, ‘vitellogenesis [GO:0007296]’ and ‘egg coat formation [GO:0035803]’. Cellular Component GO terms were screened using terms such as ‘egg coat [GO:0035805]’, ‘acrosomal vesicle [GO:0001669]’, ‘sperm flagellum [GO:0036126]’, ‘cilium [GO:0005929]’, ‘axoneme [GO:0005930]’, ‘centriole [GO:0005814]’, and ‘egg plasma membrane [GO:0001890]’. Anchor GO terms such as ‘hormone activity [GO:0005179]’, ‘steroid binding [GO:0005496]’, ‘lipoprotein receptor activity [GO:0008035]’ ‘structural constituent of egg coat [GO:0035804]’, ‘hormone receptor binding [GO:0051427]’, ‘zona pellucida receptor activity [GO:0008317]’, and ‘prostaglandin-endoperoxide synthase activity [GO:0004666]’ were used to screen for Molecular Function annotations.

Protein groups detected in at least two of the three biological replicates and in fewer than two replicates in the opposite sex were classified as sex-associated candidates. Sensitivity analysis using a stricter detection threshold requiring presence in all three biological replicates of one sex and in no more than one replicate of the other sex yielded a reduced candidate set. All proteins identified under the stricter criterion were also identified using the primary ≥2/3 replicate rule, indicating strong consistency between the two approaches and supporting the robustness of the exploratory classification strategy (Supplementary Table S1). Differential abundance analysis between male and female samples was conducted in R Studio using the ‘limma’ package. Protein group LFQ intensities were modelled using a contrast-based linear framework. Protein groups showing |log_2_ fold change| ≥ 1 and an adjusted *p*-value (Benjamini-Hochberg FDR < 0.05) were considered differentially abundant. Given the exploratory nature of this discovery proteomics dataset and the limited sample size (*n* = 3 per sex), this rule was applied as a preliminary screening approach intended to identify candidates for future investigative works rather than being definitive of sex-specific biomarkers.

## 3. Results

MaxQuant analysis of the LC-MS/MS data resulted in the assignment of 1801 protein groups based on database-matched peptide evidence against the barramundi reference database created from UniProt. Leading protein entries within each protein group, representing inferred proteoforms, were mapped using UniProt’s ID Mapping platform and were classified into their functional groups based on GO.

### 3.1. Proteome Mapping of Barramundi with Gene Ontology Functional Groups

**Figure 1:**
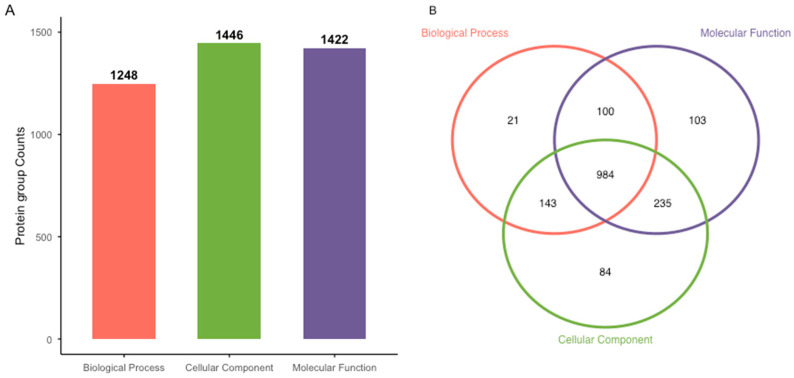
Protein group assignments derived from LC-MS/MS analysis of skin mucus from male and female barramundi. Protein groups inferred from database-matched peptide evidence were functionally annotated using GO terms across the categories Biological Process, Cellular Component, and Molecular Function (**A**). Overlap analysis illustrates the distribution of protein groups assigned to one or more GO functional categories, including protein groups assigned exclusively or jointly across GO categories (**B**).

GO-based functional annotation assigned 1248 protein groups to the Biological Process category, 1446 protein groups to Cellular Components, and 1422 protein groups to Molecular Functions (Figure 1A). Of these, 984 protein groups were matched across all three GO categories. Additionally, 143 protein groups were shared between Biological Process and Cellular Component categories, 100 protein groups between Biological Process and Molecular Function categories, and 235 protein groups between Molecular Function and Cellular Component categories (Figure 1B). A smaller number of protein groups were assigned exclusively to individual GO categories, with 21 protein groups associated only with Biological Processes, 103 protein groups only with Molecular Functions, and 84 protein groups only with Cellular Components (Figure 1B).

**Figure 2:**
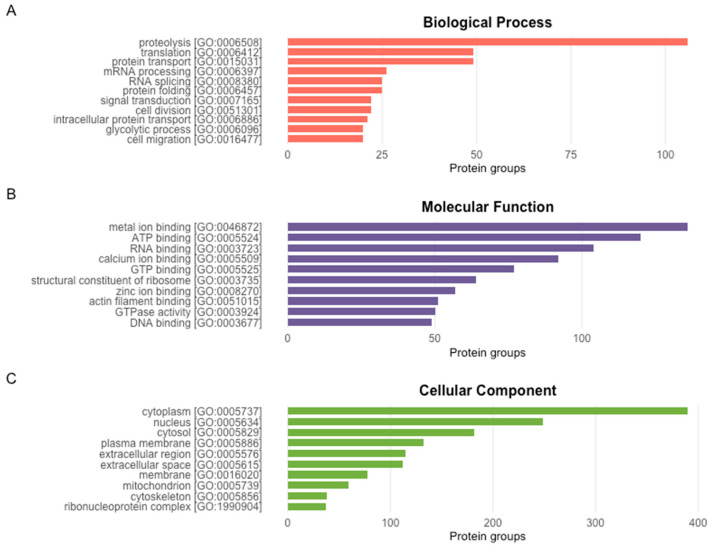
The ten most frequently assigned GO terms within each functional category—Biological Process (**A**), Molecular Function (**B**), and Cellular Component (**C**)—for protein groups inferred from LC-MS/MS analysis of barramundi skin mucus.

The top 10 most frequently represented GO terms within each functional category were extracted to summarise the dominant functional annotations assigned to protein groups inferred from barramundi skin mucus (Figure 2). Within the Biological Process category (Figure 2A), protein groups were most commonly assigned with processes including proteolysis, translation and signal transduction, indicating enrichment of functions associated with cellular communication and protein turnover. In the Molecular Function category (Figure 2B), the most frequently assigned terms included ATP binding and metal ion binding, amongst other binding activities, indicating enzyme and energy-dependent proteins. Within the Cellular Component (Figure 2C), protein groups were predominantly assigned to intracellular compartments such as the cytoplasm, nucleus, cytosol, and plasma membrane. There was also considerable representation of protein groups associated with the ribosome, cytoskeleton, and extracellular region.

### 3.2. COG Functional Classification and KEGG Pathway Mapping of Protein Groups

**Figure 3:**
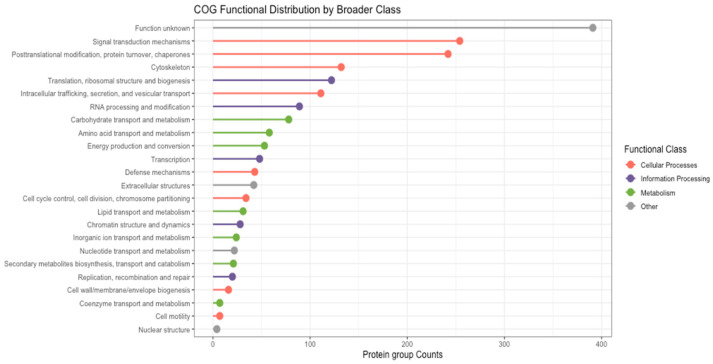
COG functional classification of protein groups inferred from LC-MS/MS analysis of barramundi skin mucus. Protein group counts are shown across COG functional categories on the y-axis and grouped by major functional classes as indicated in the legend.

COG functional classification assigned 1486 protein groups to one or more COG categories, while 391 protein groups could not be assigned a COG function. Protein groups were broadly distributed across three major COG functional classes: cellular processes and signalling, information storage and processing, and metabolism. Most of the protein groups were assigned to COG categories associated with cellular processes and signalling, including signal transduction mechanisms, post-translational modification and protein turnover, and cytoskeletal organisation (Figure 3). Protein groups assigned to metabolic COG categories were primarily associated with transport, metabolic processes, and energy conversion. Fewer protein groups were assigned to information storage and processing categories, with annotations related to translation, transcription, replication, recombination, and DNA repair (Figure 3). Overall, the COG functional distribution indicates that protein groups inferred from barramundi skin mucus span a broad range of functional classes, including structural maintenance, metabolic regulation, and defence-associated processes, reflecting the functional diversity represented within the mucus proteome.

**Figure 4:**
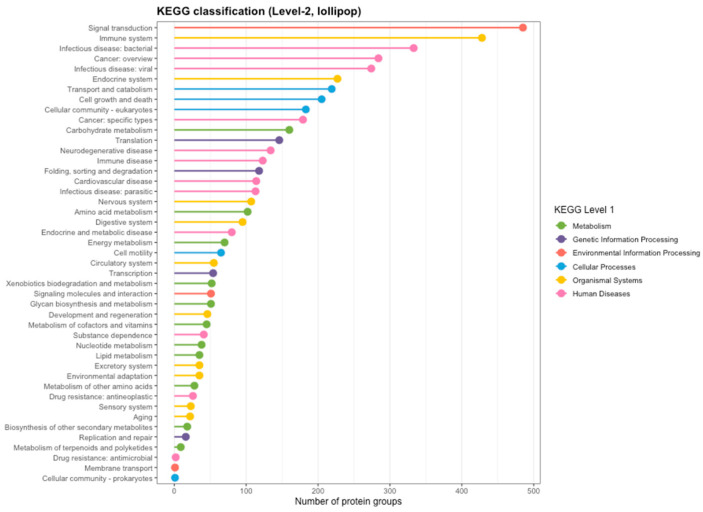
KEGG level 1 pathway classification of protein groups inferred from LC-MS/MS analysis of barramundi skin mucus.

KEGG pathway mapped assigned protein groups inferred from barramundi skin mucus to six broad level 1 categories - metabolism, genetic information processing, environmental information processing, cellular processes, organismal systems and human diseases (Figure 4). Protein groups were most frequently assigned to pathways within the organismal systems category, with prominent representation in pathways associated with immune and endocrine functions. Within the environmental information processing category, protein groups were commonly assigned to signal transduction and cellular signalling pathways. A substantial number of protein groups were also assigned to KEGG pathways classified under the human diseases category. These assignments reflect conserved molecular pathways shared across eukaryotes rather than species-specific disease states, with protein groups mapping to pathways related to bacterial, viral, and parasitic infection, as well as cancer-associated pathway frameworks (Figure 4).

### 3.3. Immune-Relevant Protein Groups in Barramundi Skin Mucus

**Figure 5:**
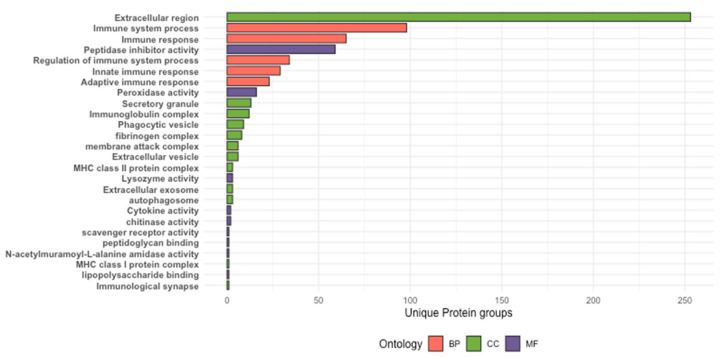
Immune-associated GO annotations among protein groups inferred from LC-MS/MS analysis of barramundi skin mucus. Protein groups prioritised by GO anchor term screening are shown across the three GO domains: Biological Process, Cellular Component, and Molecular Function.

Screening of barramundi skin mucus protein groups for immune relevance using curated Gene Ontology anchor terms prioritised 352 protein groups with immune-associated annotations. Of these, 249 protein groups were associated with Biological Processes, 318 protein groups with Cellular Component terms, and 92 protein groups with immune-associated Molecular Functions (Figure 5). Immune-associated protein groups were most prominently represented within the Cellular Component category, with frequent assignment to extracellular regions and immunoglobulin-related complexes (Figure 5). Within the Biological Process category, protein groups were commonly assigned to immune system processes and immune response-related pathways. Protein groups assigned to immune-associated Molecular Functions were predominantly linked to peptidase and peptidase inhibitor activities (Figure 5).

Across the prioritised set, multiple protein groups repeatedly associated with innate immune defence were observed based on functional annotation patterns (Supplementary Table S2). These included protein groups annotated to the complement system (e.g., C1q domain-containing entries; C3/C4/C5-associated proteins; and terminal complement pathway components C6-C9), as well as protein groups containing immunoglobulin-like domains and proteins involved in antigen presentation and immune signalling (e.g., beta-2-microglobulin, CD74 invariant chain-annotated entries) (Supplementary Table S2). Protease regulation was also prominent, with multiple protein groups annotated as serpins (e.g., Serpin B/C/D/F/G clades), cystatins, and Kunitz-type protease inhibitors, consistent with the strong representation of peptidase-inhibitor molecular functions in the GO screening (Supplementary Table S2). Additional immune-associated protein groups included lectin-related annotations (e.g., C-type lectins, galectins) and antimicrobial/recognition-associated proteins (e.g., lysozyme, peptidoglycan recognition protein annotations), supporting the breadth of immune-associated functional classes represented within the mucus proteome (Supplementary Table S2).

Integration of GO-annotated protein groups with eggNOG-based functional classification and KEGG pathway mapping further indicated that a subset of immune-associated protein groups aligned with COG categories related to defence mechanisms and KEGG pathway classes, including immune system, infectious disease, and immune disease frameworks (Supplementary Table S2). Examples included protein groups annotated as mucin-like proteins (e.g., mucin-2-like, mucin-5AC, mucin-5B) and multiple serpin/cystatin-family protease inhibitors, which were frequently assigned to immune-related KEGG pathway classes (Supplementary Table S2). Conversely, several GO-annotated immune-associated protein groups mapped to KEGG categories outside classical immune system groupings (e.g., endocrine- or digestive-associated pathway frameworks), reflect the multifunctionality and pathway overlap inherent to ontology- and homology-based annotation. Likewise, some protein groups annotated to immune-associated KEGG frameworks were not assigned to COG “defence mechanisms” categories, including complement-associated entries and immunoglobulin-like domain-containing proteins (Supplementary Table S2). Collectively, these annotation patterns highlight a broad set of putatively immune-relevant protein groups in barramundi skin mucus while remaining consistent with inference-based functional assignment.

### 3.4. Reproduction-Relevant and Sex-Associated Protein Groups in Barramundi Skin Mucus

**Figure 6:**
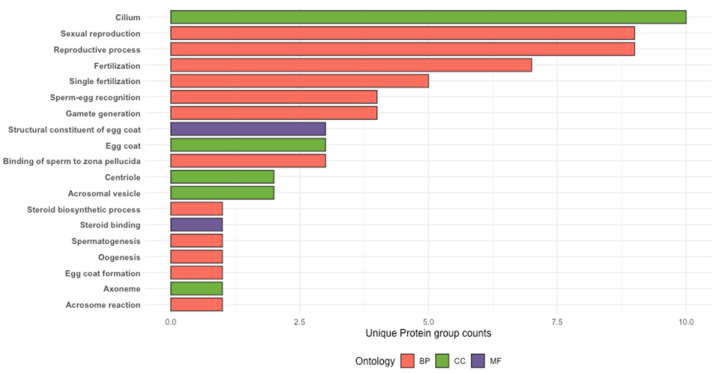
Reproduction- and sex-associated GO annotations among protein groups inferred from LC-MS/MS analysis of barramundi skin mucus. Protein groups prioritised by GO anchor term screening are shown across Biological Process, Cellular Component, and Molecular Function categories.

Screening of barramundi skin mucus protein groups for reproduction and sex relevance using curated GO anchor terms prioritised 24 protein groups with annotations linked to reproductive and sex-related processes. Across GO domains, 46 protein groups were assigned to Biological Process terms, 18 protein groups were associated with relevant Cellular Component terms, and four protein groups were assigned to Molecular Function annotations (Figure 6).

Functional integration with COG and KEGG pathway annotations indicated variable coverage among the prioritised protein groups (Supplementary Table S3). The majority of protein groups were assigned to broad COG functional classes, including signal transduction mechanisms, cytoskeletal organisation, intracellular trafficking, and lipid transport and metabolism. Representative examples included zona pellucida sperm-binding protein annotated entries, which were commonly associated with signal transduction-related COG categories, START-domain-containing protein groups associated with lipid transport and metabolic functions, and septin-annotated protein groups associated with cytoskeletal organisation and cell division-related COG classes (Supplementary Table S3). In contrast, KEGG pathway mapping was available for only a subset of the reproduction and sex-associated protein groups, with assignments primarily falling under signalling molecule and interaction frameworks and related pathway classes (Supplementary Table S3). Several protein groups lacked KEGG Orthology (KO) assignments, likely reflecting limited functional characterisation or incomplete pathway representation for reproduction-associated proteins in non-model teleost species. Collectively, these annotation patterns highlight a set of putatively reproduction-relevant and sex-associated protein groups present in barramundi skin mucus while underscoring the constraints of pathway-based annotation for reproductive biology in discovery proteomics datasets.

### 3.5. Sex-Specific Protein Differences in Barramundi Skin Mucus

Of the 1801 protein groups inferred from LC-MS/MS analysis of barramundi skin mucus, 1151 protein groups were shared between both males and females, having been detected in at least two of the three biological replicates per group. In addition, 298 protein groups were detected predominantly in male samples, and 108 protein groups were detected predominantly in female samples, based on a replicate-supported detection criterion requiring presence in at least two individuals of one sex and absence in at least two individuals of the opposite sex (Supplementary Table S4). An additional 244 protein groups did not meet the minimum replicate threshold in either group and were excluded from sex-associated comparisons. Among protein groups detected predominantly in male barramundi skin mucus, several entries, including versican core protein, START-domain-containing protein groups, septin-associated protein groups, and kinesin-like protein groups, were also prioritised during GO screening for reproduction-relevant and sex-associated annotations (Supplementary Table S3). In contrast, among protein groups detected predominantly in female barramundi skin mucus, poly (ADP-ribose) polymerase (PARP)-annotated protein groups were the only entries that overlapped with the GO-based reproduction-relevant and sex-associated screening (Supplementary Table S3). Notably, vitellogenin, a classic marker of female reproductive status, was not detected in the mucus samples analysed in this study. This absence may reflect the reproductive stage of the sampled broodstock, as vitellogenin expression is typically associated with vitellogenic females, or may indicate limited transfer of circulating vitellogenin into the mucus matrix. These sex-associated detection patterns reflect replicate-supported differences in protein group presence across male and female skin mucus samples and provide a complementary perspective to the functional annotation-based screening of reproduction- and sex-associated protein groups.

#### Differential Abundance of Proteins Between Male and Female Barramundi

**Figure 7:**
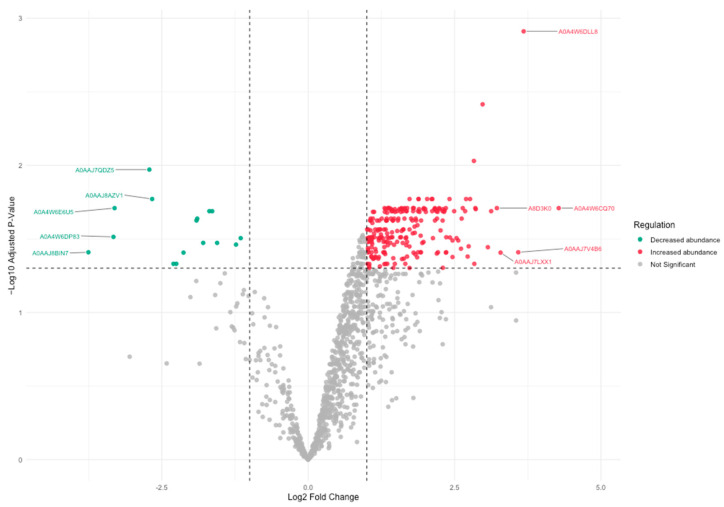
Differential abundance analysis of protein groups between male and female barramundi skin mucus samples based on LFQ intensity data. Protein groups exhibiting statistically significant differences in relative abundance are shown in red (increased abundance) and green (decreased abundance), while protein groups not meeting the significance threshold (dashed lines) are shown in grey. The five protein groups with the largest positive and negative log_2_ fold changes are annotated with their corresponding UniProt accession numbers.

Of the 1151 protein groups detected in both male and female barramundi skin mucus samples, 244 protein groups exhibited statistically significant differences in relative abundance between sexes based on LFQ intensity analysis (Figure 7; Supplementary Table S5). Of these, 228 protein groups showed increased relative abundance in males, while 16 protein groups showed decreased relative abundance. Among the protein groups displaying increased relative abundance, the five highest-ranking differentially abundant protein groups included entries annotated as 15-hydroxyprostaglandin dehydrogenase, apolipoprotein, uncharacterised oxidoreductase YjmC-like, inter-alpha-trypsin inhibitor heavy chain H3, and keratin, type I cytoskeletal 18 (Figure 7). Protein groups exhibiting the strongest decreases in relative abundance included annotations corresponding to high mobility group nucleosome-binding domain-containing protein 3, leukocyte elastase inhibitor (Serpin B1/B6), and microfibril-associated/mono (ADP-ribosyl) transferase-annotated entries (Figure 7). Integration of the differential abundance analysis with GO-based screening for reproduction-relevant and sex-associated annotations revealed that three differentially abundant protein groups, namely zona pellucida sperm-binding protein 3, sex hormone-binding globulin, and dihydrolipoyl dehydrogenase, overlapped with the reproduction-relevant and sex-associated protein group set (Supplementary Table S3). Among these, the protein group annotated as zona pellucida sperm-binding protein 3 showed decreased relative abundance in male samples, whereas protein groups annotated as sex hormone-binding globulin and dihydrolipoyl dehydrogenase showed increased relative abundance. Full statistical results, including log_2_ fold changes and significance values, are provided in Supplementary Table S5.

## 4. Discussion

This study presents the first large-scale characterisation of the barramundi skin mucus proteome using LC-MS/MS. Using a label-free, data-dependent acquisition DDA workflow, 1801 protein groups were assigned based on peptide-spectrum matching and database-driven inference, reflecting putative proteoform diversity within the mucus layer. Functional annotation using Gene Ontology, Cluster of Orthologous Groups, and Kyoto Encyclopaedia of Genes and Genomes frameworks revealed a complex molecular landscape encompassing structural, metabolic, immune, and signalling-related protein groups. These findings are consistent with reports from other teleost species, including Atlantic salmon, gilthead sea bream, and European sea bass, where similar classes of cytoskeletal and mucus-associated protein groups, lectin-related proteins, and complement-associated protein groups have been described in skin mucus [22,46,47]. Together, these results support the concept that fish skin mucus represents a multifunctional secretion involved in barrier integrity, host–environment interactions, and immune surveillance.

Fish skin mucus constitutes the first line of defence against environmental stressors and pathogens and plays a central role in mucosal immunity. GO-based screening revealed a substantial subset of protein groups assigned to immune-associated biological processes, cellular compartments, and molecular functions, with strong representation of extracellular and immunoglobulin-related components. Protein groups annotated as complement-associated components (including entries corresponding to C1q, C3/C5-related proteins, and terminal complement pathway components C6-C9) were repeatedly assigned across immune-related GO categories, consistent with their established roles in opsonisation, inflammation, and pathogen lysis [22,31]. Comparable complement-associated protein groups have been reported in the mucus of Atlantic salmon, large yellow croaker, and red tilapia [48,49,50], suggesting conservation of these innate immune mechanisms across teleosts. In addition, multiple lectin-associated protein groups, including entries corresponding to C-type lectins, were assigned, supporting their conserved function as pattern recognition molecules in mucosal defence [51]. Protein groups annotated with immunoglobulin-like domains, as well as entries corresponding to calreticulin, anti-proteases, and proteases, further indicate that barramundi skin mucus contains molecular components capable of immune modulation, antimicrobial activity, and tissue maintenance.

Barramundi are protandrous hermaphrodites, transitioning from male to female as part of their reproductive life-strategy [7]. This reproductive strategy presents challenges for sex identification and broodstock management, where sex determination often relies on invasive techniques such as gonadal cannulation or biopsy [9,10]. Sex transition can be influenced by ecological factors such as population density [52], and there is plasticity in individuals with variation in size, weight and other phenotypic characteristics, making it challenging in an aquaculture setting to appropriately maintain stocking densities for breeding. Recent studies have shown differences in sex transition between populations, with some populations demonstrating sex transition much earlier than the 3–5 years maturation age previously described [53], making it harder to select male broodstock [10]. Identifying the sex of broodstock is critical, especially since many commercially important fish are either protandrous, such as gilthead sea bream and common snook or protogynous, such as coral trout and Malabar grouper [54]. Non-invasive sampling of mucus offers a practical alternative to invasive gonadal biopsies, reducing stress and improving animal welfare. The detection of reproduction-relevant and sex-associated protein groups in skin mucus suggests that this external secretion reflects underlying physiological and endocrine states linked to sexual differentiation. GO-based screening identified a limited but informative set of protein groups associated with reproductive processes, including entries annotated to protein groups corresponding to zona pellucida sperm-binding proteins, septins, START-domain-containing proteins, and sex hormone-binding globulin. These protein groups were primarily assigned to cellular components associated with gamete structure and motility, as well as biological processes related to gametogenesis and fertilisation. Previous studies have predominantly focused on gonadal proteomics for sex identification [55]; our results demonstrate that sex-related molecular signatures are also detectable in the mucus, opening avenues for external, animal welfare-friendly diagnostic applications.

Sex-based comparisons further revealed pronounced differences in relative protein group abundance between male and female mucus samples. Although a larger number of protein groups were uniquely detected in males than in females, differential abundance analysis of shared protein groups proved more informative. Of the 1151 protein groups detected in both sexes, 244 exhibited statistically significant differences in LFQ-based relative abundance. Several protein groups overlapping with reproduction-associated GO categories also emerged as differentially abundant, including entries annotated as corresponding to zona pellucida sperm-binding protein 3, sex hormone-binding globulin, and dihydrolipoyl dehydrogenase. The absence of vitellogenin highlights the complexity of interpreting reproductive signals from mucus proteomes. Proteins detected in mucus may reflect selective transport from plasma, local epithelial secretion, or environmental exposure, and therefore may not directly mirror circulating reproductive biomarkers. These observations suggest that sex-based abundance patterns, rather than sex-exclusive presence or absence, may represent more robust molecular indicators of reproductive status in skin mucus. Because this study analysed a small number of biological replicates (*n* = 3 per sex) using a discovery-based LC-MS/MS workflow, the observed sex-associated detection patterns should be interpreted as exploratory rather than definitive indicators of sex-specific molecular signatures. In protandrous species such as barramundi, physiological and molecular differences between males and females may also reflect differences in maturation stage or endocrine status rather than strictly binary sex effects. Consequently, the sex-associated protein groups identified here should be considered candidate indicators requiring validation in larger cohorts across multiple reproductive stages before robust biological inference can be made.

The presence of multiple mucin-associated protein groups, including entries corresponding to Mucin-2, Mucin-5AC, and Mucin-5B, underscores the structural and protective functions of the mucus layer. However, mucins are highly glycosylated proteins, and their extensive post-translational modification presents inherent challenges for bottom-up proteomic analysis [56]. In this study, no enzymatic deglycosylation was performed prior to digestion; therefore, the peptide coverage and protein group assignments for mucins are likely biased toward non-glycosylated or poorly glycosylated regions. As a result, mucin-related protein groups should be interpreted cautiously, and their apparent abundance or sex association may not fully reflect the native glycoprotein complexity present in vivo. Future studies incorporating targeted glycoproteomic workflows or deglycosylation strategies would be required to more accurately characterise mucin proteoforms and their functional roles in barramundi skin mucus.

While this study demonstrates the potential of skin mucus proteomics as a non-invasive tool for health and reproductive monitoring, several methodological limitations must be considered. Blank swab controls were not included during sampling in this study, and while care was taken to minimise environmental or handling contamination, the absence of processed blank swabs means that low-abundance background proteins originating from sampling materials or environmental exposure cannot be completely excluded. Future studies should incorporate procedural blank controls to better characterise potential background signals and further refine the interpretation of mucus proteomic datasets. Nevertheless, all the detected protein groups corresponded to annotated barramundi proteins in the reference proteome, suggesting that the dataset predominantly reflects host-derived mucus proteins rather than exogenous contaminants. LC-MS/MS-based proteomics, particularly using DDA workflows, is inherently subject to stochastic precursor selection, which can lead to missing values and false-negative or false-positive protein group assignments. An additional limitation of the present study is that each biological sample was analysed using a single LC-MS/MS injection without technical replicates. Replicate injections and independent digestion replicates can reduce instrument-specific variability and stochastic peptide sampling inherent to data-dependent acquisition workflows. Consequently, the presence or absence of low-abundance proteins in this dataset should be interpreted cautiously, as some proteins may fall below detection thresholds in individual runs. Despite this limitation, the study provides a valuable first reference atlas of the barramundi skin mucus proteome and highlights candidate immune- and reproduction-associated proteins that warrant targeted validation in future studies incorporating technical replication and larger sample sizes.

Protein inference is database-dependent, and the use of a UniProt-derived barramundi reference proteome may bias assignments toward well-annotated orthologues, while underrepresenting species-specific or low-abundance proteins. Moreover, peptide-spectrum matches do not constitute analytical identification of intact proteins or true proteoforms; rather, the protein groups reported here represent inferred molecular entities derived from shared peptide evidence rather than direct identification of intact proteins or discrete proteoforms. Consequently, functional annotations and biological interpretations should be viewed as putative and hypothesis-generating rather than definitive. The relatively small sample size (*n* = 3 per sex) further limits statistical power and generalisability, despite low within-group variability (coefficient of variation < 10%). Biological heterogeneity, variation in mucus secretion, and sampling-related differences may also influence detected protein group profiles. Expanding future studies to include larger cohorts, multiple developmental stages, and environmentally relevant stressors will be essential for refining sex-specific baselines and validating candidate molecular indicators. Future work should focus on validating candidate biomarkers identified in this discovery dataset. Targeted proteomic approaches such as parallel reaction monitoring (PRM) or selected reaction monitoring (SRM) could be used to quantify selected protein groups across larger cohorts. Complementary orthogonal validation approaches, including immunoassays such as ELISA or Western blotting, would further confirm the presence and abundance of specific proteins. Expanding sampling to include different maturity stages, environmental conditions, and pathogen challenge scenarios would also allow assessment of the stability and biological relevance of candidate biomarkers for aquaculture monitoring applications. Importantly, because bottom-up proteomics relies on peptide-to-protein inference, the protein groups reported here represent putative molecular entities inferred from peptide evidence rather than direct identification of intact proteins and therefore should be interpreted as hypothesis-generating observations requiring targeted validation. Integration of complementary approaches, including transcriptomics and post-translational modification analyses, will improve confidence in protein group assignments and enhance biomarker specificity in future studies.

In summary, this study establishes a foundational reference dataset for the barramundi skin mucus proteome and demonstrates that immune-associated, reproduction-related, and sex-biased protein groups can be detected using non-invasive sampling. While the results highlight the diagnostic potential of mucus proteomics in aquaculture, careful interpretation and methodological refinement are required before translation into applied health or reproductive monitoring tools. As aquaculture advances toward precision and welfare-focused management, skin mucus proteomics represents a promising, but still developing, platform for non-lethal surveillance of fish health and physiology.

## 5. Conclusions

This study provides the first large-scale characterisation of the skin mucus proteome of barramundi and demonstrates that sex-biased differences in relative protein group abundance can be detected using non-invasive sampling. Using LC-MS/MS-based proteomics, protein groups representing inferred proteoform diversity were functionally annotated, revealing immune-associated, structural, metabolic, and reproduction-related molecular signatures within the mucus layer. The detection of immune and reproduction-associated protein groups, together with sex-biased abundance patterns, highlights the potential utility of skin mucus as a complementary matrix for investigating fish health and physiological status in aquaculture. However, these findings should be interpreted as hypothesis-generating, given the inherent limitations of bottom-up proteomics, database-driven protein inference, and the modest sample size. Establishing robust baseline datasets across developmental stages, sexes, and environmental conditions, together with integration of complementary multi-omics and targeted validation approaches, will be essential for translating skin mucus proteomics into reliable, field-deployable tools for fish welfare, reproductive management, and sustainability monitoring in aquaculture systems.

## Data Availability

Data for this study was uploaded to ProteomeXchange via MassIVE under the identifier MSV000100616. Additional data will be made available on request.

## Supplementary Materials

The following supporting information can be downloaded at: https://www.mdpi.com/article/10.3390/proteomes14010015/s1, Figure S1: SDS-PAGE gel to visualise the protein profiles of male (lanes 3–5) and female (lanes 6–8) barramundi skin mucus extracts. Lane 1 is a negative control of 2× Lysis buffer used in the protein extraction and lane 2 is a positive control of barramundi tissue extract. Table S1: Sensitivity analysis comparing sex-associated protein group detection under primary and stricter replicate thresholds. Table S2: Immune-associated protein groups assigned from barramundi skin mucus using GO-based screening and mapped to corresponding COG categories and KEGG pathways. Table S3: Sex-associated and reproduction-related protein groups assigned from barramundi skin mucus using GO-based screening and mapped to corresponding COG categories and KEGG pathways. Table S4: Protein groups uniquely detected in the skin mucus of male and female barramundi based on replicate-filtered LC-MS/MS data. Table S5: Differentially abundant protein groups in the skin mucus of male and female barramundi determined by LFQ-based analysis with adjusted *p*-values (FDR < 0.05).
