# Supplementary material for: Proteomic Insights into the Immune and Sex-Specific Proteins in the Skin Mucus of Barramundi (Lates calcarifer)
Source: Proteomes. 2026 Mar 20;14(1):15. doi: 10.3390/proteomes14010015 (PMC13030388; doi:10.3390/proteomes14010015)
Supplement: Supplementary file 1 [file proteomes-14-00015-s001.zip › proteomes-4136741-supplementary.pdf]

Supplementary material:

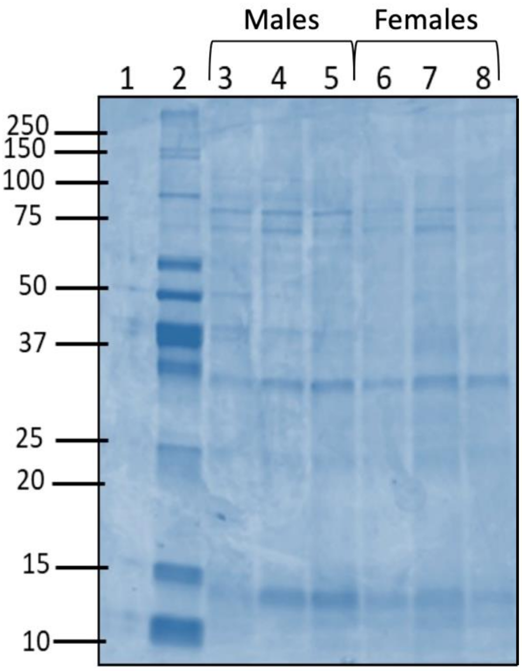

Figure S1: SDS-PAGE gel to visualise the protein profiles of male (lanes 3-5) and female (lanes 6-8) barramundi skin mucus extracts. Lane 1 is a negative control of 2x Lysis buffer used in the protein extraction and lane 2 is a positive control of barramundi tissue extract.

Table S1. Sensitivity analysis comparing sex-associated protein group detection under primary and stricter replicate thresholds.

Category	Primary detection rule (≥2/3 replicates)	Strict detection rule (3/3 replicates)	Overlap (% strict retained in primary)	Overlap (% primary retained in strict)
Male only	298	149	100	50
Female only	108	46	100	42.6

Table S2: Immune-associated protein groups assigned from barramundi skin mucus using GO based screening and mapped to corresponding COG categories and KEGG pathways.

Entry	Putative protein annotation	GO group	COG group	KEGG Level 1 term	KEGG Level 2 term
A0A4W6E1H0	60S ribosomal protein L27	BP, CC, MF	Translation, ribosomal structure and biogenesis	Genetic Information Processing	Translation
A0A4W6CRA5	Actin-related protein 2/3 complex subunit	BP, CC, MF	Cytoskeleton	Cellular Processes; Organismal Systems; Human Diseases	Transport and catabolism; Immune system; Cell motility; Infectious disease: bacterial
A0A4W6FGQ8	Activated leukocyte cell adhesion molecule b	BP, CC	Signal transduction mechanisms	Environmental Information Processing	Signaling molecules and interaction
A0A4W6CCH8<br>A0A4W6CEC6	Alpha-1-antitrypsin homolog	CC, MF	Defense mechanisms	Organismal Systems	Immune system
A0A4W6FPN5	Alpha-2-HS-glycoprotein 2	CC, MF	Signal transduction mechanisms	NA	NA
A0A4W6CME5	Alpha-2-macroglobulin-like protein 1 isoform X2	BP, CC, MF	Posttranslational modification, protein turnover, chaperones	Organismal Systems	Immune system
A0AAJ7Q8P3	Alpha-galactosidase (EC 3.2.1.-)	BP, CC, MF	Carbohydrate transport and metabolism	Metabolism; Cellular Processes	Carbohydrate metabolism; Lipid metabolism; Glycan biosynthesis and metabolism; Transport and catabolism
A0A4W6DUR7<br>A0A4W6DWE7	Amine oxidase (EC 1.4.3.-)	BP, CC, MF	Secondary metabolites biosynthesis, transport and catabolism	Metabolism	Amino acid metabolism; Biosynthesis of other secondary metabolites
A0AAJ7LV51	Aminopeptidase (EC 3.4.11.-)	BP, CC, MF	Posttranslational modification, protein turnover, chaperones	NA	NA
A0A4W6FZB7	Angiopoietin-related protein 5	BP, CC, MF	Function unknown	NA	NA
A0A4W6DNB8	Angiotensinogen (Serpin A8)	BP, CC, MF	Defense mechanisms	Organismal Systems	Endocrine system
A0A4W6CYM3	Anterior gradient 2 (Anterior gradient protein 2 homolog)	BP, CC	Function unknown	NA	NA
A0A4W6FWP4	Antithrombin-III (Serpin C1)	BP, CC, MF	Defense mechanisms	Organismal Systems; Human Diseases	Immune system; Infectious disease: parasitic
A0A4W6DST3	Apolipoprotein A-I	BP, CC, MF	Intracellular trafficking, secretion, and vesicular transport	Organismal Systems; Human Diseases	Endocrine system; Digestive system; Infectious disease: parasitic
A0A4W6DAJ1	Apolipoprotein A-IV b, tandem duplicate 2	BP, CC, MF	Signal transduction mechanisms	Organismal Systems	Digestive system

A0A4W6DAT6	Apolipoprotein C-I	BP, CC, MF	Cell wall/membrane/envelope biogenesis	Organismal Systems	Digestive system
A0A4W6DAT2	Apolipoprotein Eb	BP, CC, MF	Signal transduction mechanisms	Organismal Systems; Human Diseases	Digestive system; Neurodegenerative disease
A0A4W6FS44<br>A0A4W6FSI6<br>A0A4W6FTC3	Apolipoprotein M	CC	Function unknown	NA	NA
A0A4W6F648	Apolipoprotein M	CC	Not mapped	Not mapped	Not mapped
A0A4W6BZ98	Apoptosis-associated speck-like protein containing a CARD (PYD and CARD domain containing)	BP, CC	Function unknown	Cellular Processes; Organismal Systems; Human Diseases	Cell growth and death; Immune system; Infectious disease: bacterial; Infectious disease: viral
A0A4W6DF79	Apple domain-containing protein	BP, CC	Amino acid transport and metabolism	Organismal Systems	Immune system
A0A4W6C3M2	ATG16 autophagy related 16-like 1 (S. cerevisiae)	BP, CC, MF	Function unknown	Cellular Processes; Organismal Systems	Transport and catabolism; Immune system
A0AAJ7PM45	B-cell lymphoma/leukemia 10 isoform X2	BP, CC, MF	Function unknown	Environmental Information Processing; Organismal Systems; Human Diseases	Signal transduction; Immune system; Infectious disease: bacterial
A0A4W6BUR8<br>A0A4W6BV93	Ba1 globin, like (Hemoglobin subunit beta-A)	BP, CC, MF	Energy production and conversion	NA	NA
A0A4W6FSV5<br>A0A4W6FUX0	Beta-2-glycoprotein 1 (Apolipoprotein H) (Beta-2-glycoprotein I)	CC, MF	Signal transduction mechanisms; Defense mechanisms	Organismal Systems	Digestive system
D2CZZ6	Beta-2-microglobulin	BP, CC	Signal transduction mechanisms	Organismal Systems	Immune system
A0A4W6EPJ3	Beta-glucuronidase (EC 3.2.1.31)	BP, CC, MF	Carbohydrate transport and metabolism	Metabolism; Cellular Processes	Carbohydrate metabolism; Glycan biosynthesis and metabolism; Metabolism of cofactors and vitamins; Biosynthesis of other secondary metabolites; Xenobiotics biodegradation and metabolism; Transport and catabolism
A0AAJ7PKC6	Beta-hexosaminidase (EC 3.2.1.52)	BP, CC, MF	Carbohydrate transport and metabolism	Metabolism; Cellular Processes	Glycan biosynthesis and metabolism; Transport and catabolism
A0AAJ7PHK5	Beta-microseminoprotein-like	CC	Function unknown	NA	NA

A0A4W6EIL6	BPTI/Kunitz inhibitor domain-containing protein	BP, CC, MF	Posttranslational modification, protein turnover, chaperones	Human Diseases	Cancer: overview; Cancer: specific types
A0AAJ8DL26	Butyrophilin-like protein 1 isoform X3	BP, CC, MF	Signal transduction mechanisms	NA	NA
A0A4W6CKG3<br>A0A4W6E6X6	C-type lectin domain family 3 member B (Tetranectin)	BP, CC, MF	Signal transduction mechanisms; Defense mechanisms	NA	NA
A0A4W6E461<br>A0A4W6F4X9	C1q domain-containing protein	CC	Function unknown	NA	NA
A0A4W6D5J8	C3/C5 convertase	BP, CC, MF	Extracellular structures	Organismal Systems; Human Diseases	Immune system; Infectious disease: bacterial; Immune disease
A0A4W6CUY1<br>A0A4W6CV49<br>A0AAJ7QGR4	Calpastatin (Calpain inhibitor)	CC, MF	Function unknown	NA	NA
A0A4W6DVC4<br>A0A4W6G232<br>A0AAJ7LWW9	Calreticulin	BP, CC, MF	Posttranslational modification, protein turnover, chaperones	Genetic Information Processing; Cellular Processes; Organismal Systems; Human Diseases	Folding, sorting and degradation; Transport and catabolism; Immune system; Infectious disease: parasitic; Infectious disease: viral
A0AAJ7Q319	Calumenin-A isoform X2	CC, MF	Signal transduction mechanisms	NA	NA
A0A4W6DD56	Calumenin-B	CC, MF	Signal transduction mechanisms	NA	NA
A0A4W6CHF3	Carbonic anhydrase 6 (EC 4.2.1.1) (Carbonate dehydratase VI) (Carbonic anhydrase VI)	CC, MF	Inorganic ion transport and metabolism	Metabolism	Energy metabolism
A0AAJ7L8B5	Carboxypeptidase B2	BP, CC, MF	Posttranslational modification, protein turnover, chaperones	Environmental Information Processing; Cellular Processes; Organismal Systems; Human Diseases	Signal transduction; Cell growth and death; Circulatory system; Development and regeneration; Immune system; Environmental adaptation; Nervous system; Sensory system; Endocrine system; Endocrine and metabolic disease; Digestive system; Substance dependence; Infectious disease: bacterial; Cancer: overview; Cancer: specific types
A0A4W6EJA6	Caspase-3 (EC 3.4.22.56)	BP, CC, MF			

A0AAJ7QKI9			Cell cycle control, cell division, chromosome partitioning	Human Diseases; Environmental Information Processing; Cellular Processes; Organismal Systems	Drug resistance: antineoplastic; Signal transduction; Cell growth and death; Immune system; Nervous system; Endocrine and metabolic disease; Neurodegenerative disease; Infectious disease: bacterial; Infectious disease: parasitic; Infectious disease: viral; Cancer: overview; Cancer: specific types; Cardiovascular disease
A0A4W6FTY6	Cathepsin F (EC 3.4.22.41)	BP, CC, MF	Posttranslational modification, protein turnover, chaperones	Cellular Processes	Transport and catabolism; Cell growth and death
A0A4W6EIR0	CD44 antigen (GP90 lymphocyte homing/adhesion receptor) (HUTCH-I) (Hermes antigen) (Hyaluronate receptor) (Phagocytic glycoprotein 1) (Phagocytic glycoprotein I)	BP, CC, MF	Signal transduction mechanisms	Environmental Information Processing; Organismal Systems; Human Diseases	Signaling molecules and interaction; Immune system; Infectious disease: bacterial; Infectious disease: viral; Cancer: overview
A0A4W6F875	CD74 molecule, major histocompatibility complex, class II invariant chain b	BP, CC, MF	Function unknown	NA	NA
A0AAJ7QMI8	CD99 molecule	BP, CC	Function unknown	Environmental Information Processing; Organismal Systems	Signaling molecules and interaction; Immune system
A0A4W6F1C0	Coagulation factor IX (EC 3.4.21.22) (Christmas factor)	BP, CC, MF	Signal transduction mechanisms	Organismal Systems	Immune system
A0A4W6EYY4	Cofilin-1	BP, CC, MF	Cytoskeleton	Organismal Systems; Cellular Processes; Human Diseases	Development and regeneration; Immune system; Cell motility; Infectious disease: bacterial
A0A4W6E3T1	Collagen, type I, alpha 1b	BP, CC, MF	Extracellular structures	Environmental Information Processing; Cellular Processes; Organismal Systems; Human Diseases	Signal transduction; Cellular community - eukaryotes; Signaling molecules and interaction; Immune system; Endocrine system; Endocrine and metabolic disease; Digestive system; Infectious disease: parasitic; Infectious disease: viral
A0A4W6ESR3	Collagen, type I, alpha 2	BP, CC, MF	Extracellular structures	Environmental Information Processing; Cellular Processes;	Signal transduction; Cellular community - eukaryotes; Signaling molecules and interaction; Immune

				Organismal Systems; Human Diseases	system; Endocrine system; Endocrine and metabolic disease; Digestive system; Infectious disease: parasitic; Infectious disease: viral
A0AAJ7VHR8	Collagenase 3	BP, CC, MF	Posttranslational modification, protein turnover, chaperones	Organismal Systems; Environmental Information Processing; Human Diseases	Immune system; Signal transduction; Endocrine system; Cancer: overview; Cancer: specific types; Immune disease
A0AAJ7PIA0	Complement C1q and tumor necrosis factor-related protein 9	CC	Extracellular structures	NA	NA
A0A4W6FQ53	Complement C1q subcomponent subunit C	BP, CC	Extracellular structures	Organismal Systems; Human Diseases	Immune system; Neurodegenerative disease; Infectious disease: bacterial; Infectious disease: parasitic; Immune disease
A0AAJ7PZ14	Complement C1q tumor necrosis factor-related protein 3	CC	Function unknown	NA	NA
A0A4W6CRA0	Complement C1q-like protein 2	CC	Function unknown	NA	NA
A0AAJ7VAB9	Complement C1q-like protein 4	CC	Function unknown	NA	NA
A0AAJ8B6R0	Complement C3 isoform X1	CC, MF	Posttranslational modification, protein turnover, chaperones	Cellular Processes; Organismal Systems; Human Diseases	Transport and catabolism; Immune system; Infectious disease: bacterial; Infectious disease: parasitic; Infectious disease: viral; Cancer: overview; Immune disease
A0AAJ8DP80	Complement C3 isoform X1	CC, MF	Posttranslational modification, protein turnover, chaperones	Cellular Processes; Organismal Systems; Human Diseases	Transport and catabolism; Immune system; Infectious disease: bacterial; Infectious disease: parasitic; Infectious disease: viral; Cancer: overview; Immune disease
A0A4W6D2J4	Complement C4B (Chido/Rodgers blood group)	BP, CC, MF	Posttranslational modification, protein turnover, chaperones	Cellular Processes; Organismal Systems; Human Diseases	Transport and catabolism; Immune system; Infectious disease: bacterial; Infectious disease: parasitic; Infectious disease: viral; Cancer: overview; Immune disease
A0A4W6FLR7	Complement C5	CC, MF	Posttranslational modification, protein turnover, chaperones	Cellular Processes; Organismal Systems; Human Diseases	Transport and catabolism; Immune system; Neurodegenerative disease; Infectious disease: bacterial; Infectious disease: parasitic; Infectious disease: viral; Cancer: overview; Immune disease

A0A4W6C0D8	Complement C8 alpha chain (Complement component C8 alpha chain)	BP, CC	Signal transduction mechanisms	Organismal Systems; Human Diseases	Immune system; Neurodegenerative disease; Infectious disease: parasitic; Immune disease
A0A4W6EIF5	Complement component 1 Q subcomponent-binding protein, mitochondrial	BP, CC	Defense mechanisms	Human Diseases	Infectious disease: viral
A0A4W6CUY2	Complement component 1, r subcomponent	BP, CC, MF	Signal transduction mechanisms	Cellular Processes; Organismal Systems; Human Diseases	Transport and catabolism; Immune system; Infectious disease: bacterial; Immune disease
A0A4W6E4X1	Complement component 6, duplicate 1 (Complement component C6 isoform X1)	BP, CC	Extracellular structures	Organismal Systems; Human Diseases	Immune system; Neurodegenerative disease; Immune disease
A0A4W6FNH1	Complement component 8, gamma polypeptide (Complement component C8 gamma chain)	BP, CC, MF	Function unknown	Environmental Information Processing; Genetic Information Processing; Cellular Processes; Organismal Systems; Human Diseases	Signal transduction; Folding, sorting and degradation; Cell growth and death; Development and regeneration; Immune system; Endocrine system; Endocrine and metabolic disease; Neurodegenerative disease; Infectious disease: parasitic; Infectious disease: viral; Cancer: overview; Cancer: specific types; Immune disease
A0A4W6DQT7	Complement component c3a, duplicate 5	CC, MF	Posttranslational modification, protein turnover, chaperones	Cellular Processes; Organismal Systems; Human Diseases	Transport and catabolism; Immune system; Infectious disease: bacterial; Infectious disease: parasitic; Infectious disease: viral; Cancer: overview; Immune disease
A0A4W6EED3<br>A0A4W6EIU1	Complement component c3b, tandem duplicate 2	CC, MF	Posttranslational modification, protein turnover, chaperones	Cellular Processes; Organismal Systems; Human Diseases	Transport and catabolism; Immune system; Infectious disease: bacterial; Infectious disease: parasitic; Infectious disease: viral; Cancer: overview; Immune disease
A0A4W6E4S5	Complement component C7	BP, CC	Signal transduction mechanisms	Organismal Systems; Human Diseases	Immune system; Neurodegenerative disease; Immune disease
A0A4W6BXE3	Complement component C8 beta chain (Complement component 8 subunit beta)	BP, CC	Signal transduction mechanisms	Organismal Systems; Human Diseases	Immune system; Neurodegenerative disease; Infectious disease: parasitic; Immune disease

A0A4W6F8X5	Complement component C9	BP, CC	Signal transduction mechanisms	Organismal Systems; Human Diseases	Immune system; Neurodegenerative disease; Infectious disease: parasitic; Immune disease
A0A4W6F8J6	Complement factor H-related protein 3 isoform X1	BP, CC, MF	Signal transduction mechanisms	NA	NA
A0AAJ8B9Y2	Coxsackievirus and adenovirus receptor homolog isoform X4	BP, CC, MF	Signal transduction mechanisms	NA	NA
A0A4W6C977	creatine kinase (EC 2.7.3.2)	BP, CC, MF	Energy production and conversion	Metabolism	Amino acid metabolism
A0A4W6CZ38	CTCK domain-containing protein	CC	Function unknown	NA	NA
A0AAJ7LG25	Cystatin C (Amyloid angiopathy and cerebral hemorrhage)	CC, MF	Posttranslational modification, protein turnover, chaperones	Organismal Systems	Digestive system
A0A4W6EZ17	Cystatin F	BP, CC, MF	Posttranslational modification, protein turnover, chaperones	NA	NA
A0A4W6BWP4	Cystatin fetuin-A-type domain-containing protein	CC, MF	Function unknown	NA	NA
A0AAJ7LQ94	Cystatin-A	CC, MF	Function unknown	NA	NA
A0A4W6DL72<br>A0A4W6DL93<br>A0A4W6E496	Cystatin-B (Stefin-B)	BP, CC, MF	Function unknown	NA	NA
A0AAJ7PCB0	D-dopachrome decarboxylase (EC 4.1.1.84)	BP, CC, MF	Defense mechanisms	Metabolism	Amino acid metabolism
A0AAJ7LB66	DCC-interacting protein 13-alpha (Adapter protein containing PH domain, PTB domain and leucine zipper motif 1)	BP, CC	Signal transduction mechanisms	Organismal Systems; Human Diseases	Aging; Cancer: overview; Cancer: specific types
A0AAJ7PH23	Deoxynucleoside triphosphate triphosphohydrolase SAMHD1	BP, CC, MF	Function unknown	Genetic Information Processing	Transcription
A0AAJ7PIS1	Deoxynucleoside triphosphate triphosphohydrolase SAMHD1-like isoform X3	BP, CC, MF	Function unknown	Genetic Information Processing	Transcription
A0AAJ7LZL5	Dermatopontin-like	BP, CC	Not mapped	Not mapped	Not mapped
A0A4W6E287	Di-N-acetylchitobiase	BP, CC, MF	Carbohydrate transport and metabolism	NA	NA

A0A4W6DNW2	Dihydrolipoyl dehydrogenase (EC 1.8.1.4)	BP, CC, MF	Energy production and conversion	Metabolism	Carbohydrate metabolism; Amino acid metabolism
A0A4W6FPE1	Dipeptidyl peptidase 2 (EC 3.4.14.2) (Dipeptidyl aminopeptidase II) (Dipeptidyl peptidase 7) (Dipeptidyl peptidase II) (Quiescent cell proline dipeptidase)	BP, CC, MF	Posttranslational modification, protein turnover, chaperones	Organismal Systems	Endocrine system; Digestive system
A0AAJ8AWC0	E3 ubiquitin-protein ligase Midline-1 isoform X2	BP, CC, MF	Posttranslational modification, protein turnover, chaperones	Environmental Information Processing; Organismal Systems; Human Diseases	Signal transduction; Immune system; Infectious disease: viral
A0AAJ7V761	Ectonucleotide pyrophosphatase/phosphodiesterase family member 2 isoform X1	BP, CC, MF	Function unknown	Metabolism	Lipid metabolism
A0A4W6GCI6	Eosinophil peroxidase	BP, CC, MF	Function unknown	Metabolism; Cellular Processes; Organismal Systems; Human Diseases	Amino acid metabolism; Xenobiotics biodegradation and metabolism; Transport and catabolism; Endocrine system; Cancer: overview; Cancer: specific types; Immune disease
A0A4W6D839<br>A0A4W6FNU4	Ependymin	BP, CC, MF	Function unknown	NA	NA
A0A4W6E375<br>A0AAJ7PWN7	Extracellular matrix protein 1	BP, CC	Function unknown	NA	NA
A0A4W6BQJ2	F5/8 type C domain-containing protein	BP, MF	Function unknown	NA	NA
A0A4W6D1J1	Fas (tnfrsf6)-associated via death domain (Protein FADD)	BP, CC, MF	Signal transduction mechanisms	Human Diseases; Cellular Processes; Organismal Systems; Environmental Information Processing	Drug resistance: antineoplastic; Cell growth and death; Immune system; Signal transduction; Neurodegenerative disease; Infectious disease: parasitic; Infectious disease: bacterial; Infectious disease: viral; Cancer: overview
A0A4W6F7K4	Fetuin B	BP, CC, MF	Function unknown	NA	NA
A0A4W6F7K9	Fetuin-B	CC, MF	Function unknown	NA	NA
A0A4W6CP46<br>A0AAJ7LD03	Fibrinogen alpha chain	BP, CC, MF	Function unknown	Organismal Systems; Human Diseases	Immune system; Infectious disease: bacterial
A0A4W6CPK0	Fibrinogen beta chain	BP, CC, MF	Function unknown	Organismal Systems	Immune system

A0A4W6BPQ2	Fibrinogen gamma chain	BP, CC, MF	Function unknown	Organismal Systems; Human Diseases	Immune system; Infectious disease: bacterial
A0AAJ8B728	Fibrinogen gamma chain isoform X2	BP, CC, MF	Function unknown	Organismal Systems; Human Diseases	Immune system; Infectious disease: bacterial
A0A4W6EMU3	Fibroblast growth factor binding protein 1b (Fibroblast growth factor-binding protein 1)	BP, CC, MF	Function unknown	NA	NA
A0A4W6CVV3	Fibulin-1	BP, CC, MF	Signal transduction mechanisms	NA	NA
A0A4W6DSK6	FinTRIM family, member 86	BP, CC, MF	Posttranslational modification, protein turnover, chaperones	Environmental Information Processing; Organismal Systems; Human Diseases	Signal transduction; Immune system; Infectious disease: viral
A0AAJ7LKE3	FYN-binding protein 1 isoform X3	BP, CC	Signal transduction mechanisms	NA	NA
A0A4W6BSF7	G3BP stress granule assembly factor 2b	BP, CC, MF	RNA processing and modification	NA	NA
A0A4W6ECL6	Galectin	CC, MF	Extracellular structures	NA	NA
A0A4W6G5S8	Galectin	BP, CC, MF	Extracellular structures	NA	NA
A0A4W6F7N5	Gamma-interferon-inducible lysosomal thiol reductase (EC 1.8.-.-) (Gamma-interferon-inducible protein IP-30)	BP, CC, MF	Posttranslational modification, protein turnover, chaperones	Organismal Systems	Immune system
A0A4W6FDV0	Gelsolin (ADF) (Actin-depolymerizing factor)	BP, CC, MF	Cytoskeleton	Organismal Systems; Cellular Processes; Human Diseases	Immune system; Cell motility; Cancer: overview
A0A4W6DM07	GH18 domain-containing protein	BP, CC, MF	Carbohydrate transport and metabolism	Metabolism	Glycan biosynthesis and metabolism
A0A4W6DWW0	Glutaminyl-peptide cyclotransferase (EC 2.3.2.5) (Glutaminyl cyclase)	CC, MF	Posttranslational modification, protein turnover, chaperones	NA	NA
A0AAJ7PMZ8	Glutathione peroxidase	BP, CC, MF	Posttranslational modification, protein turnover, chaperones	Metabolism; Cellular Processes; Organismal Systems	Metabolism of other amino acids; Lipid metabolism; Cell growth and death; Endocrine system
A0A4W6D2N4<br>A0AAJ7LUV6<br>A0AAJ7PYL4	Glutathione peroxidase	BP, CC, MF	Posttranslational modification, protein turnover, chaperones	Metabolism; Organismal Systems	Metabolism of other amino acids; Lipid metabolism; Endocrine system
A0A4W6D9X9	Glutathione S-transferase kappa (EC 2.5.1.18)	BP, CC, MF	Carbohydrate transport and metabolism	Metabolism; Cellular Processes; Human Diseases	Metabolism of other amino acids; Xenobiotics biodegradation and

					metabolism; Transport and catabolism; Cancer: overview
A0AAJ7QMU6	Glypican-1	BP, CC, MF	Signal transduction mechanisms	Human Diseases	Cancer: overview; Cardiovascular disease
A0AAJ7LK17	Golgi-associated plant pathogenesis-related protein 1 isoform X1	CC	Function unknown	NA	NA
A0A4W6E7M6	Granulin b	BP, CC	Signal transduction mechanisms	NA	NA
A0A4W6CN21	GTPase activating protein (SH3 domain) binding protein 1	BP, CC, MF	RNA processing and modification	NA	NA
A0AAJ7VA46	H-2 class II histocompatibility antigen, E-D beta chain	BP, CC	Function unknown	Cellular Processes; Environmental Information Processing; Organismal Systems; Human Diseases	Transport and catabolism; Signaling molecules and interaction; Immune system; Endocrine and metabolic disease; Infectious disease: parasitic; Infectious disease: bacterial; Infectious disease: viral; Immune disease; Cardiovascular disease
A0A4W6EWH0	Haptoglobin	BP, CC, MF	Amino acid transport and metabolism	NA	NA
A0A4W6F1K2	Heat shock protein 90, alpha (cytosolic), class A member 1, tandem duplicate 2	BP, CC, MF	Posttranslational modification, protein turnover, chaperones	Genetic Information Processing; Environmental Information Processing; Cellular Processes; Organismal Systems; Human Diseases	Folding, sorting and degradation; Signal transduction; Cell growth and death; Immune system; Environmental adaptation; Endocrine system; Cancer: overview; Cancer: specific types; Cardiovascular disease
A0A4W6BV66	Hemoglobin subunit alpha	BP, CC, MF	Energy production and conversion	Human Diseases	Infectious disease: parasitic
A0A4W6C1K8<br>A0AAJ7QK86	Hemopexin	BP, CC, MF	Posttranslational modification, protein turnover, chaperones; Extracellular structures	NA	NA
A0AAJ7VI41	Hepatocyte growth factor-like protein	BP, CC, MF	Signal transduction mechanisms	NA	NA
A0AAJ7QHR1	High mobility group protein B2 (High mobility group protein 2)	BP, CC, MF	Transcription	NA	NA

A0AAJ7LRA0	HLA class II histocompatibility antigen, DP alpha 1 chain	BP, CC	Signal transduction mechanisms	Cellular Processes; Environmental Information Processing; Organismal Systems; Human Diseases	Transport and catabolism; Signaling molecules and interaction; Immune system; Endocrine and metabolic disease; Infectious disease: parasitic; Infectious disease: bacterial; Infectious disease: viral; Immune disease; Cardiovascular disease
A0A4W6G9L5	Hyaluronan and proteoglycan link protein 1	BP, CC, MF	Signal transduction mechanisms	NA	NA
A0AAJ8DT66	Ig heavy chain Mem5	BP, CC	Function unknown	NA	NA
A0A4W6CY96<br>A0A4W6ECY8<br>A0A4W6EJP9<br>A0A4W6ELJ2<br>A0A4W6EM85<br>A0A4W6EMK6<br>A0A4W6EPP5<br>A0A4W6EQR0	Ig-like domain-containing protein	BP, CC	Function unknown	NA	NA
A0A4W6F8N5	Ig-like domain-containing protein	BP, CC, MF	Signal transduction mechanisms	NA	NA
A0A4W6F9K7	Ig-like domain-containing protein	BP, CC	Signal transduction mechanisms	NA	NA
A0A4W6EPU4<br>A0A4W6ESX3<br>A0A4W6ET05	Ig-like domain-containing protein	BP, CC	Function unknown	Environmental Information Processing; Cellular Processes; Organismal Systems; Human Diseases	Signal transduction; Transport and catabolism; Immune system; Infectious disease: parasitic; Infectious disease: bacterial; Infectious disease: viral; Cancer: overview; Immune disease; Cardiovascular disease
A0A4W6D4E2<br>A0A4W6FME3	Ig-like domain-containing protein	BP, CC	Function unknown	Cellular Processes; Environmental Information Processing; Organismal Systems; Human Diseases	Transport and catabolism; Cell growth and death; Signaling molecules and interaction; Immune system; Endocrine and metabolic disease; Infectious disease: viral; Cancer: overview; Immune disease; Cardiovascular disease
A0AAJ7LGI9	IgGFc-binding protein	CC	Defense mechanisms; Extracellular structures	NA	NA

A0A4W6BKV2	ILEI/PANDER domain-containing protein	CC, MF	Function unknown	NA	NA
A0AAJ7Q216	Insulin-like growth factor-binding protein 7	BP, CC, MF	Signal transduction mechanisms	NA	NA
A0A4W6EYC6	Integrin alpha-6 isoform X2 (Integrin, alpha 6b)	BP, CC, MF	Extracellular structures	Environmental Information Processing; Cellular Processes; Organismal Systems; Human Diseases	Signal transduction; Cellular community - eukaryotes; Signaling molecules and interaction; Immune system; Cell motility; Infectious disease: parasitic; Infectious disease: viral; Cancer: overview; Cancer: specific types; Cardiovascular disease
A0A4W6D833	Inter-alpha-trypsin inhibitor heavy chain 2	BP, CC, MF	Function unknown	NA	NA
A0A4W6EX30	Inter-alpha-trypsin inhibitor heavy chain 3b, tandem duplicate 1	CC, MF	Function unknown	NA	NA
A0AAJ7V4B6	Inter-alpha-trypsin inhibitor heavy chain H3	BP, CC, MF	Function unknown	NA	NA
A0A4W6G1N1	Interferon regulatory factor 6	BP, CC, MF	Function unknown	NA	NA
A0A4W6E300	Interferon-induced protein 35	BP, CC	Function unknown	NA	NA
A0A4W6DBA3	Intermediate filament protein ON3 (Keratin 5)	BP, CC, MF	Function unknown	NA	NA
A0AAJ7PFF3	Intestinal mucin-like protein	CC	Defense mechanisms; Extracellular structures	Environmental Information Processing; Cellular Processes; Organismal Systems; Human Diseases	Signal transduction; Cellular community - eukaryotes; Signaling molecules and interaction; Immune system; Digestive system; Infectious disease: parasitic; Infectious disease: viral; Cancer: specific types
A0A4W6C5Y7	Keratin 4	BP, CC, MF	Function unknown	NA	NA
A0A4W6DAF5<br>A0A4W6DAZ7<br>A0A4W6DBM8	Keratin 5	BP, CC, MF	Function unknown	NA	NA
A0AAJ7LVH9	Keratin, type II cytoskeletal 8	BP, CC, MF	Function unknown	NA	NA
A0AAJ7PXZ7	Keratin, type II cytoskeletal 8 isoform X2	BP, CC, MF	Function unknown	NA	NA
A0AAJ7LNV5	Kininogen-1 isoform X2	BP, CC, MF	Posttranslational modification, protein turnover, chaperones	Organismal Systems	Immune system

A0AAJ7QNL9	Kunitz-type protease inhibitor 2	CC, MF	Posttranslational modification, protein turnover, chaperones	Organismal Systems	Immune system
A0A4W6C5Q1	Laminin, gamma 1	BP, CC	Extracellular structures	Environmental Information Processing; Cellular Processes; Human Diseases	Signal transduction; Cellular community - eukaryotes; Signaling molecules and interaction; Neurodegenerative disease; Infectious disease: parasitic; Infectious disease: viral; Cancer: overview; Cancer: specific types
A0A4W6EKF1	Large ribosomal subunit protein eL22 (60S ribosomal protein L22)	BP, CC, MF	Translation, ribosomal structure and biogenesis	Genetic Information Processing	Translation
A0AAJ7QJB2	Latexin isoform X2	CC, MF	Function unknown	NA	NA
A0AAJ7LN82	Leukocyte elastase inhibitor (Serpin B1)	CC, MF	Defense mechanisms	Human Diseases	Infectious disease: parasitic
A0A4W6E6Z2<br>A0AAJ8AZQ0<br>A0AAJ8AZV1	Leukocyte elastase inhibitor (Serpin B1) (Serpin B6)	CC, MF	Defense mechanisms	Human Diseases	Infectious disease: parasitic
A0AAJ7VDX8<br>A0AAJ8DJW2	LOW QUALITY PROTEIN: alpha-2-macroglobulin-like	BP, CC, MF	Posttranslational modification, protein turnover, chaperones	Organismal Systems	Immune system
A0AAJ8DQS6	LOW QUALITY PROTEIN: apolipoprotein B-100-like	BP, CC, MF	Lipid transport and metabolism	Organismal Systems	Digestive system
A0AAJ8B9I2	LOW QUALITY PROTEIN: apolipoprotein Bb, tandem duplicate 1	BP, CC, MF	Lipid transport and metabolism	Organismal Systems	Digestive system
A0AAJ8BCM0	LOW QUALITY PROTEIN: collagen alpha-5(IV) chain-like	CC	Function unknown	Organismal Systems; Human Diseases	Immune system; Neurodegenerative disease; Infectious disease: bacterial; Infectious disease: parasitic; Immune disease
A0AAJ7PPP5	LOW QUALITY PROTEIN: complement C3-like	CC, MF	Posttranslational modification, protein turnover, chaperones	Cellular Processes; Organismal Systems; Human Diseases	Transport and catabolism; Immune system; Infectious disease: bacterial; Infectious disease: parasitic; Infectious disease: viral; Cancer: overview; Immune disease

A0AAJ8B8Z7	LOW QUALITY PROTEIN: complement factor I-like	BP, CC, MF	Posttranslational modification, protein turnover, chaperones	Organismal Systems; Human Diseases	Immune system; Infectious disease: bacterial
A0AAJ7PM17	LOW QUALITY PROTEIN: fucolectin-7-like	BP, CC, MF	Function unknown	NA	NA
A0AAJ7PW98	LOW QUALITY PROTEIN: granulin a	CC	Signal transduction mechanisms	NA	NA
A0AAJ7QN90	LOW QUALITY PROTEIN: hemoglobin subunit alpha-A-like	BP, CC, MF	Energy production and conversion	Human Diseases	Infectious disease: parasitic
A0AAJ7PFD3	LOW QUALITY PROTEIN: intestinal mucin-like protein	CC	Defense mechanisms; Extracellular structures	Environmental Information Processing; Cellular Processes; Organismal Systems; Human Diseases	Signal transduction; Cellular community - eukaryotes; Signaling molecules and interaction; Immune system; Digestive system; Infectious disease: parasitic; Infectious disease: viral; Cancer: specific types
A0AAJ7Q3M3	LOW QUALITY PROTEIN: kunitz-type protease inhibitor 1a	BP, CC, MF	Posttranslational modification, protein turnover, chaperones	Human Diseases	Cancer: overview; Cancer: specific types
A0AAJ8B4E1	LOW QUALITY PROTEIN: mucin-2-like	CC	Defense mechanisms; Extracellular structures	Environmental Information Processing; Cellular Processes; Organismal Systems; Human Diseases	Signal transduction; Cellular community - eukaryotes; Signaling molecules and interaction; Immune system; Digestive system; Infectious disease: parasitic; Infectious disease: viral; Cancer: specific types
A0AAJ8DMN1	LOW QUALITY PROTEIN: mucin-5AC	CC	Defense mechanisms; Extracellular structures	Environmental Information Processing; Cellular Processes; Organismal Systems; Human Diseases	Signal transduction; Cellular community - eukaryotes; Signaling molecules and interaction; Immune system; Digestive system; Infectious disease: parasitic; Infectious disease: viral; Cancer: specific types
A0AAJ7VLC8	LOW QUALITY PROTEIN: phosphatidylethanolamine-binding protein 1	BP, CC, MF	Function unknown	Organismal Systems	Immune system
A0AAJ8DM39	LOW QUALITY PROTEIN: uncharacterized protein LOC108893007	BP, CC	Amino acid transport and metabolism	Organismal Systems	Immune system

A0A4W6E3V7	Lumican	CC	Signal transduction mechanisms	Human Diseases	Cancer: overview
A0A4W6G3E6	Lysine--tRNA ligase (EC 6.1.1.6) (Lysyl-tRNA synthetase)	BP, CC, MF	Translation, ribosomal structure and biogenesis	Genetic Information Processing	Translation
M1S1U1	lysozyme (EC 3.2.1.17)	BP, MF	Carbohydrate transport and metabolism	Organismal Systems	Digestive system
A8D3J6	Lysozyme g (EC 3.2.1.17)	BP, CC, MF	Carbohydrate transport and metabolism	NA	NA
A0A4W6EPI0	Macrophage migration inhibitory factor (EC 5.3.2.1) (EC 5.3.3.12) (L-dopachrome isomerase) (L-dopachrome tautomerase) (Phenylpyruvate tautomerase)	BP, CC, MF	Defense mechanisms	Metabolism	Amino acid metabolism
A0AAJ7PZQ8	Mamu class II histocompatibility antigen, DR alpha chain	BP, CC	Signal transduction mechanisms	Cellular Processes; Environmental Information Processing; Organismal Systems; Human Diseases	Transport and catabolism; Signaling molecules and interaction; Immune system; Endocrine and metabolic disease; Infectious disease: parasitic; Infectious disease: bacterial; Infectious disease: viral; Immune disease; Cardiovascular disease
A0A4W6EL15	MANSC domain-containing protein	BP, CC, MF	Posttranslational modification, protein turnover, chaperones	Human Diseases	Cancer: overview; Cancer: specific types
A0A4W6E222	Matrix metallopeptidase 30	BP, CC, MF	Posttranslational modification, protein turnover, chaperones	Organismal Systems; Environmental Information Processing; Human Diseases	Immune system; Signal transduction; Cancer: overview; Cancer: specific types; Immune disease
A0A4W6BVF3	Mesencephalic astrocyte-derived neurotrophic factor	BP, CC	Function unknown	NA	NA
A0A4W6FHV0<br>A0A4W6FHY1<br>A0AAJ7PZV0	Metalloendopeptidase (EC 3.4.24.-)	BP, CC, MF	Posttranslational modification, protein turnover, chaperones	NA	NA
A0A4W6DSF0	Metalloproteinase inhibitor 2 (Tissue inhibitor of metalloproteinases 2)	BP, CC, MF	Posttranslational modification, protein turnover, chaperones	NA	NA
A0A4W6EBM7	Microfibril associated protein 4	BP, CC, MF	Function unknown	NA	NA

A0A4W6E6U5	Microfibril associated protein 4 (Microfibril-associated glycoprotein 4)	BP, CC, MF	Function unknown	NA	NA
A0AAJ7V6Q2	Mucin-2	CC	Defense mechanisms; Extracellular structures	Environmental Information Processing; Cellular Processes; Organismal Systems; Human Diseases	Signal transduction; Cellular community - eukaryotes; Signaling molecules and interaction; Immune system; Digestive system; Infectious disease: parasitic; Infectious disease: viral; Cancer: specific types
A0AAJ8BH02	Mucin-5AC	CC	Defense mechanisms; Extracellular structures	Environmental Information Processing; Cellular Processes; Organismal Systems; Human Diseases	Signal transduction; Cellular community - eukaryotes; Signaling molecules and interaction; Immune system; Digestive system; Infectious disease: parasitic; Infectious disease: viral; Cancer: specific types
A0AAJ8BB58<br>A0AAJ8BMG9	Mucin-5B	CC	Defense mechanisms; Extracellular structures	Environmental Information Processing; Cellular Processes; Organismal Systems; Human Diseases	Signal transduction; Cellular community - eukaryotes; Signaling molecules and interaction; Immune system; Digestive system; Infectious disease: parasitic; Infectious disease: viral; Cancer: specific types
A0AAJ7LR88	Myeloid-derived growth factor	BP, CC	Function unknown	NA	NA
A0AAJ7QEK8	Myosin-9 (Cellular myosin heavy chain, type A) (Myosin heavy chain 9) (Myosin heavy chain, non-muscle IIa) (Non-muscle myosin heavy chain A) (Non-muscle myosin heavy chain IIa)	BP, CC, MF	Cytoskeleton	Cellular Processes; Organismal Systems; Human Diseases	Cell growth and death; Circulatory system; Cellular community - eukaryotes; Cardiovascular disease
A0AAJ7PEF6	Myosin-9 (Myosin heavy chain 9) (Myosin heavy chain, non-muscle IIa) (Non-muscle myosin heavy chain IIa)	BP, CC, MF	Cytoskeleton	Cellular Processes	Cellular community - eukaryotes
A0A4W6FP12	N-acetylmuramoyl-L-alanine amidase (Peptidoglycan recognition protein 2)	BP, MF	Cell wall/membrane/envelope biogenesis	NA	NA

A0A4W6CZ50	NACHT, LRR and PYD domains-containing protein 1a	BP, CC	Function unknown	Cellular Processes; Organismal Systems; Human Diseases	Cell growth and death; Immune system; Infectious disease: bacterial; Infectious disease: viral
A0A4W6E467	NAD(P)H-hydrate epimerase (EC 5.1.99.6) (Apolipoprotein A-I-binding protein) (AI-BP) (NAD(P)HX epimerase)	CC, MF	Carbohydrate transport and metabolism	NA	NA
A0A4W6CBM0	non-specific serine/threonine protein kinase (EC 2.7.11.1)	BP, CC, MF	Signal transduction mechanisms	NA	NA
A0A4W6CG49	Nucleobindin-1	CC, MF	Function unknown	NA	NA
A0A4W6F429	Optineurin	BP, CC, MF	RNA processing and modification	Cellular Processes	Transport and catabolism
A0A4W6D811	Papilin	BP, CC, MF	Signal transduction mechanisms	NA	NA
A0A4W6EQ21	Papilin b, proteoglycan-like sulfated glycoprotein	BP, CC, MF	Posttranslational modification, protein turnover, chaperones	NA	NA
A0A4W6E8H1	Pentraxin 3 (Pentraxin-related protein PTX3)	BP, CC, MF	Function unknown	NA	NA
A0A4W6DSJ5	Pentraxin family member	CC, MF	Function unknown	NA	NA
A0A4W6DTK6	Pentraxin family member	CC, MF	Function unknown	NA	NA
A0A4W6E1N1	Pentraxin family member	CC, MF	Extracellular structures	NA	NA
A0A4W6F480	Peroxiredoxin-1 (EC 1.11.1.24)	BP, CC, MF	Posttranslational modification, protein turnover, chaperones	Cellular Processes	Transport and catabolism; Cell growth and death
A0A4W6FQR7	Peroxiredoxin-1 (EC 1.11.1.24)	BP, CC, MF	Posttranslational modification, protein turnover, chaperones	Cellular Processes	Transport and catabolism; Cell growth and death
A0A4W6F0X1	Peroxiredoxin-5 (EC 1.11.1.24)	BP, CC, MF	Posttranslational modification, protein turnover, chaperones	Cellular Processes	Transport and catabolism
A0A4W6BRB8	Peroxiredoxin-6 (EC 1.11.1.27) (EC 2.3.1.23) (EC 3.1.1.4) (1-Cys peroxiredoxin) (Acidic calcium-independent phospholipase A2) (Glutathione-dependent peroxiredoxin) (Lysophosphatidylcholine acyltransferase 5) (Non-selenium glutathione peroxidase)	BP, CC, MF	Posttranslational modification, protein turnover, chaperones	Metabolism	Biosynthesis of other secondary metabolites
A0AAJ7PHV9	Peroxiredoxin-6 (EC 1.11.1.27) (EC 2.3.1.23) (EC 3.1.1.4) (1-Cys peroxiredoxin) (Acidic calcium-independent phospholipase A2) (Glutathione-dependent peroxiredoxin) (Lysophosphatidylcholine acyltransferase 5) (Non-selenium glutathione peroxidase)	BP, CC, MF	Posttranslational modification, protein turnover, chaperones	Metabolism	Biosynthesis of other secondary metabolites

A0AAJ7LL54	PG-M	BP, CC, MF	Signal transduction mechanisms	Environmental Information Processing	Signaling molecules and interaction
A0A4W6GBG9	Phosphoinositide-3-kinase-interacting protein 1	BP, CC, MF	Signal transduction mechanisms	NA	NA
A0A4W6DLI3	Phospholipase B-like (EC 3.1.1.-)	BP, CC, MF	Signal transduction mechanisms	NA	NA
A0A4W6C6V9	Pigment epithelium-derived factor	BP, CC, MF	Defense mechanisms	Environmental Information Processing	Signal transduction
A0A4W6DMG4	PITH domain-containing protein 1	BP, CC	Posttranslational modification, protein turnover, chaperones	NA	NA
A0A4W6CJ84	Plasma retinol-binding protein II	CC, MF	Function unknown	NA	NA
A0A4W6D8I6	Poly [ADP-ribose] polymerase (PARP) (EC 2.4.2.-)	BP, CC, MF	Transcription; Replication, recombination and repair; Posttranslational modification, protein turnover, chaperones	Genetic Information Processing; Cellular Processes; Organismal Systems	Replication and repair; Cell growth and death; Aging
A0A4W6D5A2	Pro-interleukin-16 [Cleaved into: Interleukin-16 (IL-16) (Lymphocyte chemoattractant factor) (LCF)]	BP, CC, MF	Function unknown	NA	NA
A0AAJ8DVV1	Prosaposin isoform X2	BP, CC	Carbohydrate transport and metabolism; Lipid transport and metabolism	Cellular Processes	Transport and catabolism
A0A4W6DCH2	Proteasome inhibitor PI31 subunit	BP, CC, MF	Posttranslational modification, protein turnover, chaperones	Genetic Information Processing	Folding, sorting and degradation
A0A4W6F0A6<br>A0A4W6F0G2<br>A0AAJ7Q4A7	Proteasome subunit beta	BP, CC, MF	Posttranslational modification, protein turnover, chaperones	Genetic Information Processing	Folding, sorting and degradation
A0AAJ7PI43	Protein AMBP	CC, MF	Posttranslational modification, protein turnover, chaperones	NA	NA

A0AAJ8DRW2	Protein AMBP isoform X4	CC, MF	Posttranslational modification, protein turnover, chaperones	NA	NA
A0AAJ7PYM2	Protein FAM3C isoform X2	CC, MF	Function unknown	NA	NA
A0AAJ8BFP1	Protein NLRC3 isoform X3	BP, CC, MF	Function unknown	NA	NA
A0A4W6FDQ8	Protein S100 (S100 calcium-binding protein)	CC, MF	Signal transduction mechanisms	NA	NA
A0AAJ7PQK1	Protein S100 (S100 calcium-binding protein)	CC, MF	Function unknown	NA	NA
A0A4W6FVK6	Protein S100-A1	CC, MF	Function unknown	NA	NA
A0AAJ7PWL0	Protein S100-A13	CC, MF	Function unknown	NA	NA
A0A4W6FBW7	Protein Z-dependent protease inhibitor (Serpin peptidase inhibitor, clade A (alpha-1 antiproteinase, antitrypsin), member 10b)	BP, CC, MF	Defense mechanisms	NA	NA
A0A4W6C042	Protein Z, vitamin K-dependent plasma glycoprotein a	BP, CC, MF	Signal transduction mechanisms	Organismal Systems	Immune system
A0A4W6ERX5<br>A0A4W6ERX9<br>A0A4W6EUL4<br>A0AAJ7LYL1	Protein-glutamine gamma-glutamyltransferase 2 (EC 2.3.2.13) (EC 3.5.1.44) (Isopeptidase TGM2) (Protein-glutamine deamidase TGM2) (Protein-glutamine dopaminyltransferase TGM2) (Protein-glutamine histaminyltransferase TGM2) (Protein-glutamine noradrenalinyltransferase TGM2) (Protein-glutamine serotonyltransferase TGM2) (Tissue transglutaminase) (Transglutaminase-2)	BP, CC, MF	Function unknown	Human Diseases	Neurodegenerative disease
A0A4W6DU43	Prothrombin (EC 3.4.21.5) (Coagulation factor II)	BP, CC, MF	Posttranslational modification, protein turnover, chaperones	Environmental Information Processing; Organismal Systems; Cellular Processes	Signaling molecules and interaction; Immune system; Cell motility

A0A4W6CP92	RAB2A, member RAS oncogene family (Ras-related protein Rab-2A)	BP, CC, MF	Intracellular trafficking, secretion, and vesicular transport	Environmental Information Processing	Signal transduction
A0A4W6FC99	RAB7a, member RAS oncogene family (Ras-related protein Rab-7a)	BP, CC, MF	Intracellular trafficking, secretion, and vesicular transport	Cellular Processes; Human Diseases	Transport and catabolism; Infectious disease: bacterial; Infectious disease: parasitic
A0A4W6CIW0<br>A0A4W6DZF2	Ras-related protein Rab-14 (EC 3.6.5.2)	BP, CC, MF	Intracellular trafficking, secretion, and vesicular transport	Environmental Information Processing	Signal transduction
A0AAJ7VHJ4	Ras-related protein Rab-3	BP, CC, MF	Intracellular trafficking, secretion, and vesicular transport	Organismal Systems	Digestive system
A0A4W6D9M0	Renin receptor (ATPase H(+)-transporting lysosomal accessory protein 2) (ATPase H(+)-transporting lysosomal-interacting protein 2) (Renin/prorenin receptor)	BP, CC, MF	Energy production and conversion	Organismal Systems	Endocrine system
A0A4W6FC31	S100 calcium binding protein U	CC, MF	Function unknown	NA	NA
A0A4W6FV78	S100 calcium binding protein W	CC, MF	Signal transduction mechanisms	NA	NA
A0A4W6F9E2	Saxitoxin and tetrodotoxin-binding protein 1	CC	Not mapped	Not mapped	Not mapped
A0A4W6EQQ4	Secreted frizzled-related protein 3 (Frizzled-related protein 1) (FrzB-1)	BP, CC, MF	Signal transduction mechanisms	NA	NA
A0AAJ7PMH9	Semaphorin-1A	BP, CC, MF	Signal transduction mechanisms	Environmental Information Processing; Organismal Systems	Signal transduction; Development and regeneration; Nervous system
A0AAJ8AYK8	Serine/threonine-protein kinase OSR1 (EC 2.7.11.1) (Oxidative stress-responsive 1 protein)	BP, CC, MF	Signal transduction mechanisms	NA	NA
A0A4W6DMW2	Serotransferrin	BP, CC, MF	Energy production and conversion	NA	NA
A0A4W6DLP4	Serotransferrin	BP, CC, MF	Inorganic ion transport and metabolism	Environmental Information Processing; Cellular Processes; Organismal Systems	Signal transduction; Cell growth and death; Digestive system
A0AAJ7QE68<br>A0AAJ7VHC8	Serpin B6	CC, MF	Defense mechanisms	Human Diseases	Infectious disease: parasitic

A0A4W6C6U3	Serpin domain-containing protein	CC, MF	Defense mechanisms	Organismal Systems	Immune system
A0AAJ7V9S5	Serpin peptidase inhibitor, clade A (Alpha-1 antiproteinase, antitrypsin), member 10a	BP, CC, MF	Defense mechanisms	NA	NA
A0A4W6BRV5	Serpin peptidase inhibitor, clade D (heparin cofactor), member 1	BP, CC, MF	Defense mechanisms	Organismal Systems	Immune system
A0A4W6BUX6	Serpin peptidase inhibitor, clade F (Alpha-2 antiplasmin, pigment epithelium derived factor), member 2b	CC, MF	Defense mechanisms	Organismal Systems	Immune system
A0A4W6BR61	Serpin peptidase inhibitor, clade G (C1 inhibitor), member 1	CC, MF	Defense mechanisms	Organismal Systems; Human Diseases	Immune system; Infectious disease: bacterial
A0A4W6CUF5	Sex hormone-binding globulin	CC, MF	Signal transduction mechanisms	NA	NA
A0A4W6DHN3	Si:ch211-175m2.5	MF	Function unknown	NA	NA
A0A4W6EVW1	Signal transducer and activator of transcription	BP, CC, MF	Transcription; Signal transduction mechanisms	Environmental Information Processing; Organismal Systems; Cellular Processes; Human Diseases	Signal transduction; Immune system; Cell growth and death; Development and regeneration; Endocrine system; Endocrine and metabolic disease; Infectious disease: parasitic; Infectious disease: bacterial; Infectious disease: viral; Cancer: overview; Cancer: specific types; Immune disease
A0A4W6FAD6	Slit homolog 3 (Drosophila)	BP, CC, MF	Signal transduction mechanisms	Organismal Systems	Development and regeneration
A0A4W6FSE2	small monomeric GTPase (EC 3.6.5.2)	BP, CC, MF	Intracellular trafficking, secretion, and vesicular transport	Organismal Systems	Digestive system
A0A4W6DJZ9	small monomeric GTPase (EC 3.6.5.2)	BP, CC, MF	Intracellular trafficking, secretion, and vesicular transport	Cellular Processes; Environmental Information Processing	Transport and catabolism; Signal transduction; Cellular community - eukaryotes
A0A4W6G4J2	small monomeric GTPase (EC 3.6.5.2)	BP, CC, MF	Intracellular trafficking, secretion, and vesicular transport	Cellular Processes; Environmental Information Processing; Organismal Systems	Transport and catabolism; Signal transduction; Cellular community - eukaryotes; Digestive system

A0A4W6BNQ4<br>A0A4W6D6I4	small monomeric GTPase (EC 3.6.5.2)	BP, CC, MF	Intracellular trafficking, secretion, and vesicular transport	Cellular Processes; Environmental Information Processing; Organismal Systems	Transport and catabolism; Signal transduction; Excretory system; Digestive system
A0A4W6E5W7	Small ribosomal subunit protein uS11 (40S ribosomal protein S14)	BP, CC, MF	Translation, ribosomal structure and biogenesis	Genetic Information Processing	Translation
A0A4W6D317	Small ribosomal subunit protein uS4 (40S ribosomal protein S9)	BP, CC, MF	Translation, ribosomal structure and biogenesis	Genetic Information Processing	Translation
A0A4W6C5D4	Small ribosomal subunit protein uS8 (40S ribosomal protein S15a)	BP, CC, MF	Translation, ribosomal structure and biogenesis	Genetic Information Processing	Translation
A0AAJ8DR46	Soluble scavenger receptor cysteine-rich domain-containing protein SSC5D	BP, CC, MF	Signal transduction mechanisms	Organismal Systems	Digestive system
A0A4W6CF37	Sorting nexin-3	BP, CC, MF	Intracellular trafficking, secretion, and vesicular transport	Cellular Processes	Transport and catabolism
A0A4W6EEM3	Spondin-1 (F-spondin)	BP, CC, MF	Extracellular structures	NA	NA
A0A4W6CDW6	ST14 transmembrane serine protease matriptase a	BP, CC, MF	Amino acid transport and metabolism	Environmental Information Processing; Organismal Systems; Human Diseases	Signaling molecules and interaction; Immune system; Digestive system; Infectious disease: viral; Cancer: overview
A0A4W6FUJ7	ST14 transmembrane serine protease matriptase b	BP, CC, MF	Amino acid transport and metabolism	Environmental Information Processing; Organismal Systems; Human Diseases	Signaling molecules and interaction; Immune system; Digestive system; Infectious disease: viral; Cancer: overview
A0AAJ7PJZ5	Sulfhydryl oxidase (EC 1.8.3.2)	BP, CC, MF	Cell cycle control, cell division, chromosome partitioning	NA	NA
A0A4W6C1S3	Superoxide dismutase [Cu-Zn] (EC 1.15.1.1)	CC, MF	Inorganic ion transport and metabolism	NA	NA
A0A4W6FUN1	Thyroxine-binding globulin (Serpin A7) (T4-binding globulin)	CC, MF	Defense mechanisms	Organismal Systems	Immune system
A0A4W6CZQ2	Toll-interacting protein	BP, CC, MF	Function unknown	Organismal Systems	Immune system
A0A4W6BMF0	Tolloid-like protein 2	CC, MF	Signal transduction mechanisms	Organismal Systems; Human Diseases	Development and regeneration; Infectious disease: viral

A0AAJ7LLA3	Transcobalamin-2	BP, CC, MF	Function unknown	Organismal Systems	Digestive system
A0AAJ7PLN1	Transcription initiation factor TFIID subunit 11	MF	Function unknown	NA	NA
A0A4W6CD81	Trimethylguanosine synthase (Cap-specific guanine-N(2) methyltransferase) (Nuclear receptor coactivator 6-interacting protein) (PRIP-interacting protein with methyltransferase motif)	BP, CC, MF	Function unknown	Genetic Information Processing; Human Diseases	Translation; Neurodegenerative disease
A0A4W6BXI8<br>A0A4W6C5W1<br>A0AAJ7LGF5<br>A0AAJ7LIF3<br>A0AAJ8BID2<br>A0AAJ8DVW3	trypsin (EC 3.4.21.4)	BP, CC, MF	Amino acid transport and metabolism	Cellular Processes; Organismal Systems; Human Diseases	Cell growth and death; Endocrine system; Immune system; Endocrine and metabolic disease; Cancer: overview; Immune disease
A0A4W6DI18	trypsin (EC 3.4.21.4)	BP, CC, MF	Posttranslational modification, protein turnover, chaperones	Organismal Systems; Human Diseases	Immune system; Infectious disease: bacterial
A0A4W6C1F9	trypsin (EC 3.4.21.4)	BP, CC, MF	Posttranslational modification, protein turnover, chaperones	Organismal Systems; Human Diseases	Immune system; Infectious disease: bacterial; Cancer: overview; Immune disease
A0A4W6C9V6	trypsin (EC 3.4.21.4)	BP, CC, MF	Signal transduction mechanisms	NA	NA
A0AAJ7LHJ4<br>A0AAJ8B0I5	trypsin (EC 3.4.21.4)	BP, CC, MF	Amino acid transport and metabolism	NA	NA
A0AAJ7QEA0	Tumor protein D52 isoform X2	BP, CC	Function unknown	NA	NA
A0A4W6FSF6	Uncharacterized protein	CC	Function unknown	NA	NA
A0A4W6FTB3	Uncharacterized protein	CC	Function unknown	NA	NA
A0A4W6FTU8	Uncharacterized protein	CC	Function unknown	NA	NA
A0A4W6CT88	Uncharacterized protein	BP, CC, MF	Extracellular structures	Environmental Information Processing; Cellular Processes; Organismal Systems; Human Diseases	Signal transduction; Cellular community - eukaryotes; Signaling molecules and interaction; Immune system; Endocrine system; Endocrine and metabolic disease; Digestive system; Infectious disease: parasitic; Infectious disease: viral

A0AAJ8DSY9	Uncharacterized protein LOC108873550 isoform X2	CC	Not mapped	Not mapped	Not mapped
A0AAJ7PEF2	Uncharacterized protein LOC108875889	CC	Function unknown	NA	NA
A0A4W6G889	Uncharacterized protein LOC108878177	CC	Not mapped	Not mapped	Not mapped
A0AAJ8DNH7	Uncharacterized protein LOC108879369 isoform X1	BP, CC	Function unknown	NA	NA
A0AAJ7LKX7	Uncharacterized protein LOC108879370	BP, CC	Not mapped	Not mapped	Not mapped
A0AAJ7PNA6	Uncharacterized protein LOC108884286 isoform X3	BP, CC, MF	Function unknown	NA	NA
A0A4W6F654	Uncharacterized protein LOC108892780	CC	Not mapped	Not mapped	Not mapped
A0A4W6G148	V-set domain containing T cell activation inhibitor 1	BP, CC, MF	Signal transduction mechanisms	Environmental Information Processing	Signaling molecules and interaction
A0A4W6G9P7<br>A0AAJ8B987	Versican core protein (Chondroitin sulfate proteoglycan core protein 2) (Large fibroblast proteoglycan) (PG-M)	BP, CC, MF	Signal transduction mechanisms	Environmental Information Processing	Signaling molecules and interaction
A0A4W6D7W0	Vigilin (High density lipoprotein-binding protein)	BP, CC, MF	Lipid transport and metabolism	NA	NA
A0A4W6CLJ3	Vitamin K-dependent protein C (EC 3.4.21.69) (Anticoagulant protein C) (Autoprothrombin IIA) (Blood coagulation factor XIV)	BP, CC, MF	Posttranslational modification, protein turnover, chaperones	Organismal Systems	Immune system
A0A4W6DD37<br>A0A4W6CZY1	VWFD domain-containing protein	CC	Defense mechanisms; Extracellular structures	Environmental Information Processing; Cellular Processes; Organismal Systems; Human Diseases	Signal transduction; Cellular community - eukaryotes; Signaling molecules and interaction; Immune system; Digestive system; Infectious disease: parasitic; Infectious disease: viral; Cancer: specific types
A0A4W6DP83	Zona pellucida sperm-binding protein 3	BP, CC, MF	Signal transduction mechanisms	NA	NA
A0A4W6E6R1<br>A0AAJ7PDS3	Zona pellucida sperm-binding protein 4 (Zona pellucida	BP, CC, MF	Signal transduction mechanisms	NA	NA

	glycoprotein 4) (Zona pellucida protein B)				

Table S3. Sex-associated and reproduction-related protein groups assigned from barramundi skin mucus using GO based screening and mapped to corresponding COG categories and KEGG pathways.

Entry	Putative protein annotation	GO group	COG group	KEGG Level 1 term	KEGG Level 2 term
A0A4W6DNW2	Dihydrolipoyl dehydrogenase (EC 1.8.1.4)	BP, CC, MF	Energy production and conversion	Metabolism	Carbohydrate metabolism; Amino acid metabolism
A0A4W6CKW6	Doublecortin domain-containing protein	BP, CC	Cell cycle control, cell division, chromosome partitioning; Cytoskeleton	NA	NA
A0AAJ8BGT4	Dynactin subunit 1	BP, CC, MF	Cell cycle control, cell division, chromosome partitioning; Cytoskeleton	Organismal Systems; Human Diseases	Excretory system; Neurodegenerative disease
A0A4W6D8I5	Golgi resident protein GCP60 (Acyl-CoA-binding domain-containing protein 3) (Golgi complex-associated protein 1) (Golgi phosphoprotein 1)	BP, CC, MF	Intracellular trafficking, secretion, and vesicular transport	NA	NA
A0A4W6C470<br>A0A4W6CKP6	Kinesin-like protein	BP, CC, MF	Cytoskeleton	Cellular Processes; Organismal Systems	Transport and catabolism; Nervous system
A0A4W6E2D8	Lymphocyte antigen-6, epidermis (Sperm acrosome membrane-associated protein 4)	BP, CC	Function unknown	NA	NA
A0AAJ7PRL8	Poly [ADP-ribose] polymerase (PARP) (EC 2.4.2.-)	BP, CC, MF	Transcription; Replication, recombination and repair; Posttranslational modification, protein turnover, chaperones	Genetic Information Processing; Cellular Processes; Organismal Systems	Replication and repair; Cell growth and death; Aging
A0A4W6FNS4	protein deglycase (EC 3.5.1.124) (Maillard deglycase)	BP, CC, MF	Defense mechanisms	Human Diseases	Neurodegenerative disease

	(Parkinsonism-associated deglycase)				
A0A4W6CP92	RAB2A, member RAS oncogene family (Ras-related protein Rab-2A)	BP, CC, MF	Intracellular trafficking, secretion, and vesicular transport	Environmental Information Processing	Signal transduction
A0A4W6FZW5<br>A0AAJ8DXI2	Septin	BP, CC, MF	Cell cycle control, cell division, chromosome partitioning; Intracellular trafficking, secretion, and vesicular transport; Cytoskeleton	NA	NA
A0A4W6CUF5	Sex hormone-binding globulin	CC, MF	Signal transduction mechanisms	NA	NA
A0A4W6CYY2	Sorbitol dehydrogenase (Polyol dehydrogenase)	BP, CC, MF	Secondary metabolites biosynthesis, transport and catabolism	Metabolism	Carbohydrate metabolism
A0A4W6CDW6	ST14 transmembrane serine protease matriptase a	BP, CC, MF	Amino acid transport and metabolism	Environmental Information Processing; Organismal Systems; Human Diseases	Signaling molecules and interaction; Immune system; Digestive system; Infectious disease: viral; Cancer: overview
A0A4W6EFX1<br>A0A4W6F3D8<br>A0A4W6BQF0	START domain-containing protein 10 (PCTP-like protein) (StAR-related lipid transfer protein 10)	BP, CC, MF	Lipid transport and metabolism	NA	NA
A0AAJ8BFX6	Suppressor of tumorigenicity 14 protein homolog	BP, CC, MF	Amino acid transport and metabolism	Environmental Information Processing; Organismal Systems; Human Diseases	Signaling molecules and interaction; Immune system; Digestive system; Infectious disease: viral; Cancer: overview
A0A4W6G9P7<br>A0AAJ8B987	Versican core protein (Chondroitin sulfate proteoglycan core protein 2) (Large fibroblast proteoglycan) (PG-M)	BP, CC, MF	Signal transduction mechanisms	Environmental Information Processing	Signaling molecules and interaction
A0A4W6DP83	Zona pellucida sperm-binding protein 3	BP, CC, MF	Signal transduction mechanisms	NA	NA

A0A4W6E6R1					
A0AAJ7PDS3	Zona pellucida sperm-binding protein 4 (Zona pellucida glycoprotein 4) (Zona pellucida protein B)	BP, CC, MF	Signal transduction mechanisms	NA	NA

Table S4: Protein groups uniquely detected in the skin mucus of male and female barramundi based on replicate-filtered LC-MS/MS data.

UniProt Accession	Putative protein annotations
**Female Only**	
A0A4W6BJH1	WASP like actin nucleation promoting factor
A0A4W6BKV2	ILEI/PANDER domain-containing protein
A0AAJ7VB10	Vesicle-associated membrane protein-associated protein B/C isoform X2
A0A4W6BNX4	Nucleophosmin
A0A4W6BP83	PDS5 cohesin associated factor A
A0A4W6BQY7	Adapter molecule crk
A0A4W6BTR7	Vesicle transport through interaction with t-SNAREs homolog 1A (Vesicle transport v-SNARE protein Vti1-like 2) (Vti1-rp2)
A0A4W6BUT8	Histone deacetylase complex subunit SAP18
A0A4W6BVS6	RAB18B, member RAS oncogene family
A0A4W6C5F8	Peptidase S1 domain-containing protein
A0A4W6C5R7	Peptidase S1 domain-containing protein
A0AAJ8DVY7	Protein phosphatase 1 regulatory subunit 12A (Myosin phosphatase-targeting subunit 1)
A0A4W6BYS8	Si:ch73-22o12.1
A0A4W6C0J3	DUF4592 domain-containing protein
A0A4W6C8I2	Gse1 coiled-coil protein b
A0A4W6DRV1	HMG box domain containing 3 (HMG domain-containing protein 3)
A0A4W6C4Q5	Acyl carrier protein
A0A4W6CHA8	Protein S100 (S100 calcium-binding protein)
A0A4W6C6U2	Neural proliferation differentiation and control protein 1a
A0AAJ7PFX4	Arrestin, beta 2a isoform X2
A0A4W6CCP6	SWI/SNF related, matrix associated, actin dependent regulator of chromatin subfamily c member 2
A0A4W6CCS7	Copine-1 isoform X1 (RNA binding motif protein 12)
A0A4W6CDI2	TAR DNA-binding protein 43
A0A4W6CIE4	Fatty acid-binding protein, liver (L-FABP) (Liver-type fatty acid-binding protein)
A0AAJ7LRR0	Copper transport protein ATOX1 (Metal transport protein ATX1)

A0A4W6CN21	GTPase activating protein (SH3 domain) binding protein 1
A0A4W6CQD2	non-specific serine/threonine protein kinase (EC 2.7.11.1)
A0AAJ7PRJ3	Coiled-coil-helix-coiled-coil-helix domain-containing protein 2
A0A4W6CRB3	Clathrin interactor 1a
A0A4W6CTI8	Carboxypeptidase (EC 3.4.16.-)
A0A4W6FEU6	Mago homolog, exon junction complex subunit
A0A4W6CWG2	RNA binding motif protein 4.3
A0A4W6D5A8	Acetyltransferase component of pyruvate dehydrogenase complex (EC 2.3.1.12)
A0A4W6D6E5	Nuclear transcription factor Y subunit gamma (CAAT box DNA-binding protein subunit C) (Nuclear transcription factor Y subunit C)
A0AAJ7QDB8	Ubiquilin-4 (Ataxin-1 interacting ubiquitin-like protein) (Ataxin-1 ubiquitin-like-interacting protein A1U) (Connexin43-interacting protein of 75 kDa) (Ubiquilin-like protein)
A0AAJ7QE38	Pyridoxal-dependent decarboxylase domain-containing protein 1
A0A4W6D8H2	Ribosomal protein S10
A0A4W6D9R4	Splicing factor, arginine/serine-rich 1
A0A4W6DA92	Tubulin beta chain
A0A4W6DAI9	Splicing factor 3b, subunit 2
A0A4W6DN64	H15 domain-containing protein
A0A4W6DSF0	Metalloproteinase inhibitor 2 (Tissue inhibitor of metalloproteinases 2)
A0A4W6DWD4	Protein scribble homolog
A0A4W6DZW9	Splicing factor 3B subunit 4
A0A4W6E0M6	Si:dkey-87o1.2
A0A4W6E3Z0	LSM12 homolog b (Protein LSM12 homolog A)
A0A4W6E6Z2	Leukocyte elastase inhibitor (Serpin B1) (Serpin B6)
A0A4W6E787	LSM14A mRNA processing body assembly factor b
A0A4W6E8V2	PDZ and LIM domain protein 4
A0AAJ7PDF5	Echinoderm microtubule-associated protein-like 4 isoform X2
A0A4W6EHP6	DENN domain containing 4C
A0A4W6EIF5	Complement component 1 Q subcomponent-binding protein, mitochondrial
A0A4W6ENE9	Nuclear transcription factor Y, beta b
A0A4W6EMM2	Lactoylglutathione lyase (EC 4.4.1.5) (Glyoxalase I)
A0A4W6EPI8	Ig-like domain-containing protein
A0A4W6EVL6	Protein disulfide-isomerase (EC 5.3.4.1)
A0AAJ8DQD9	CCHC-type zinc finger, nucleic acid binding protein a isoform X4
A0A4W6F393	Formin binding protein 1
A0A4W6EZ17	Cystatin F
A0A4W6EZC0	Cytochrome c oxidase copper chaperone
A0A4W6F1D0	Endonuclease domain-containing 1 protein
A0A4W6F1K2	Heat shock protein 90, alpha (cytosolic), class A member 1, tandem duplicate 2

A0A4W6FK87	Deoxyribonuclease I
A0AAJ7PKT0	Transcription factor BTF3
A0A4W6F4G1	Nucleophosmin 1a
A0A4W6F875	CD74 molecule, major histocompatibility complex, class II invariant chain b
A0A4W6F9B4	Keratin type 1 c19e (Keratin, type I cytoskeletal 13)
A0AAJ7Q687	Spartin
A0A4W6FDN7	RNA binding motif, single stranded interacting protein 2a (RNA-binding motif, single-stranded-interacting protein 2 isoform X1)
A0A4W6FI71	Deoxynucleotidyltransferase terminal-interacting protein 1 (Terminal deoxynucleotidyltransferase-interacting factor 1)
A8D8I2	Cathepsin L
A0A4W6FMS8	Ig-like domain-containing protein
A0A4W6FWL0	Polypyrimidine tract-binding protein 1
A0A4W6FW81	Ras-related protein Rab-15 (EC 3.6.5.2)
A0A4W6FY05	Olfactory marker protein a
A0A4W6FYD5	Cytochrome c oxidase assembly factor 6
A0A4W6FZY1	40S ribosomal protein S25
A0AAJ7PSD3	Histone H1.0-B
A0AAJ7PBV3	ATP synthase-coupling factor 6, mitochondrial (ATPase subunit F6)
A0A4W6G404	Multiple C2 domains, transmembrane 2b
A0A4W6G3L9	Scaffold attachment factor B
A0A4W6G450	Glycine cleavage system H protein
A0AAJ7LKE7	Activated RNA polymerase II transcriptional coactivator p15 (SUB1 homolog)
A0AAJ7LAV8	Ganglioside GM2 activator
A0AAJ7LJV3	Ladinin-1 isoform X3
A0AAJ7LQ21	DNA polymerase (EC 2.7.7.7)
A0AAJ7PF40	Myosin light polypeptide 6 isoform X2
A0AAJ7PIA0	Complement C1q and tumor necrosis factor-related protein 9
A0AAJ7PIT3	GRIP1-associated protein 1 isoform X3
A0AAJ7PLN1	Transcription initiation factor TFIID subunit 11
A0AAJ7PPM2	Uncharacterized protein cusr
A0AAJ7PRL8	Poly [ADP-ribose] polymerase (PARP) (EC 2.4.2.-)
A0AAJ7PWN7	Extracellular matrix protein 1
A0AAJ7Q1M6	Kinesin-like protein KIF20A isoform X3
A0AAJ7Q2M7	Uncharacterized protein LOC108892176
A0AAJ7Q693	Uncharacterized protein LOC108893845
A0AAJ7QI32	Ribosomal protein L15
A0AAJ7QKM3	D-aminoacyl-tRNA deacylase (EC 3.1.1.96)
A0AAJ8DTT6	Uncharacterized protein LOC108887798 isoform X3
A0AAJ8B0I5	trypsin (EC 3.4.21.4)

A0AAJ8B941	Hemicentin-1 isoform X32
A0AAJ8B8L4	LOW QUALITY PROTEIN: kinesin-like protein KIF13B
A0AAJ8B9A5	Protocadherin gamma-A2 isoform X40
A0AAJ8BB58	Mucin-5B
A0AAJ8BCM3	LIM domain only protein 7b isoform X7
A0AAJ8BLJ3	Fibrous sheath CABYR-binding protein
A0AAJ8DM88	Uncharacterized protein LOC108891826
A0AAJ8DN34	Titin (EC 2.7.11.1) (Connectin)
**Male Only**	
A0A4W6BPQ2	Fibrinogen gamma chain
A0A4W6BM67	Platelet-activating factor acetylhydrolase IB subunit alpha1 (EC 3.1.1.47) (PAF acetylhydrolase 29 kDa subunit) (PAF-AH subunit gamma)
A0A4W6BQB8	Potassium channel tetramerization domain containing 17
A0A4W6BNK7	Coronin
A0A4W6D5C0	G1 to S phase transition 1, like
A0A4W6BKX2	Capping protein regulator and myosin 1 linker 1 (F-actin-uncapping protein LRRC16A)
A0AAJ7LTL4	Eukaryotic translation elongation factor 1 delta a (Guanine nucleotide exchange protein) isoform X9
A0A4W6BLL5	Mitogen-activated protein kinase (EC 2.7.11.24)
A0AAJ7QND7	Prostaglandin reductase 1 (EC 1.3.1.48) (EC 1.3.1.74) (15-oxoprostaglandin 13-reductase) (Dithiolethione-inducible gene 1 protein) (Leukotriene B4 12-hydroxydehydrogenase) (NAD(P)H-dependent alkenal/one oxidoreductase)
A0A4W6BNL5	UDP-glucose 6-dehydrogenase (EC 1.1.1.22)
A0A4W6BP60	60S ribosomal protein L36a
A0A4W6C3J9	PrdX deacylase domain-containing protein 1
A0A4W6BQD0	ATP-citrate synthase (EC 2.3.3.8) (ATP-citrate (pro-S-)-lyase) (Citrate cleavage enzyme)
A0A4W6BZF0	Thimet oligopeptidase (EC 3.4.24.15)
A0AAJ7Q4A7	Proteasome subunit beta
A0A4W6BSJ1	Coronin
A0A4W6BYC5	UDP-N-acetylglucosamine pyrophosphorylase 1
A0A4W6BTC9	Ras-related protein Rab-25
A0A4W6BUL6	Purine nucleoside phosphorylase (EC 2.4.2.1) (Inosine phosphorylase) (Inosine-guanosine phosphorylase)
A0AAJ7V761	Ectonucleotide pyrophosphatase/phosphodiesterase family member 2 isoform X1
A0A4W6BUR8	Ba1 globin, like (Hemoglobin subunit beta-A)
A0A4W6BV66	Hemoglobin subunit alpha
A0A4W6BVE0	Coatomer subunit gamma
A0A4W6C470	Kinesin-like protein
A0A4W6BXE3	Complement component C8 beta chain (Complement component 8 subunit beta)
A0A4W6C6W0	NADH dehydrogenase [ubiquinone] 1 alpha subcomplex subunit 8
A0A4W6C075	Ras-related GTP-binding protein

A0A4W6C0D8	Complement C8 alpha chain (Complement component C8 alpha chain)
A0AAJ7LA17	Large ribosomal subunit protein eL28 (60S ribosomal protein L28)
A0A4W6C1V5	IQ motif containing GTPase activating protein 1
A0A4W6C1Y3	Glutamate dehydrogenase
A0A4W6CB83	5'-nucleotidase, cytosolic IIb
A0A4W6C476	Serine protease 59, putative (Thymus-specific serine protease)
A0A4W6E5B3	Adenylate cyclase-inhibiting G alpha protein
A0A4W6CCJ2	Eukaryotic translation initiation factor 4E family member 1c
A0A4W6FU68	Katanin catalytic subunit A1 like 2
A0AAJ7LVI4	H/ACA ribonucleoprotein complex subunit 2 (Nucleolar protein family A member 2)
A0A4W6C9C2	Golgi reassembly-stacking protein 2
A0AAJ7PJI1	Large ribosomal subunit protein uL11 (60S ribosomal protein L12)
A0A4W6CEQ7	Heterogeneous nuclear ribonucleoprotein M
A0A4W6CD85	Alpha-adducin (Erythrocyte adducin subunit alpha)
A0A4W6CDD9	Annexin
A0AAJ7PJB9	Ubiquitin carboxyl-terminal hydrolase (EC 3.4.19.12)
A0A4W6CDZ1	Elongin-B (Elongin 18 kDa subunit) (RNA polymerase II transcription factor SIII subunit B) (SIII p18) (Transcription elongation factor B polypeptide 2)
A0A4W6CE06	Cysteine and glycine-rich protein 1 (Cysteine-rich protein 1)
A0A4W6CF37	Sorting nexin-3
A0A4W6CF94	Ig-like domain-containing protein
A0A4W6CKP6	Kinesin-like protein
A0A4W6CID0	KH-type splicing regulatory protein
A0AAJ7PIL3	Annexin
A0A4W6CLJ3	Vitamin K-dependent protein C (EC 3.4.21.69) (Anticoagulant protein C) (Autoprothrombin IIA) (Blood coagulation factor XIV)
A0A4W6CLT4	Dual specificity mitogen-activated protein kinase kinase 2 (EC 2.7.12.2) (ERK activator kinase 2) (MAPK/ERK kinase 2)
A0A4W6CN62	Ubiquitin-like domain-containing protein
A0A4W6CNF5	Bifunctional purine biosynthesis protein ATIC (EC 2.1.2.3) (EC 3.5.4.10) (AICAR transformylase/inosine monophosphate cyclohydrolase)
A0A4W6CNT6	Mitochondrial fission 1 protein
A0A4W6CP80	Proteasome (prosome, macropain) assembly chaperone 3 (Proteasome assembly chaperone 3)
A0A4W6CPJ6	Carbonic anhydrase (EC 4.2.1.1)
A0A4W6CPS4	Pulmonary surfactant-associated protein D
A0A4W6CRA5	Actin-related protein 2/3 complex subunit
A0A4W6CRA0	Complement C1q-like protein 2
A0A4W6CR46	Tubulin polymerization-promoting protein family member 3
A0A4W6CRC8	glutathione transferase (EC 2.5.1.18)
A0A4W6CRI5	Serine/threonine-protein phosphatase (EC 3.1.3.16)

A0A4W6CRV6	Uncharacterized protein
A0A4W6CSE6	Ribosomal protein
A0A4W6CSD8	N-myc downstream regulated 1a (Protein NDRG1a)
A0A4W6CT16	H(+)-transporting two-sector ATPase (EC 7.1.2.2)
A0A4W6CT88	Uncharacterized protein
A0A4W6CT44	Bis(5'-nucleosyl)-tetraphosphatase [asymmetrical] (EC 3.6.1.17) (Diadenosine 5',5'''-P1,P4-tetraphosphate asymmetrical hydrolase) (Nucleoside diphosphate-linked moiety X motif 2)
A0AAJ7VHR8	Collagenase 3
A0A4W6CV32	Catenin alpha-1
A0AAJ7PWZ2	Arf-GAP with dual PH domain-containing protein 1 isoform X2
A0A4W6CVV3	Fibulin-1
A0A4W6CWS3	Vacuolar protein sorting-associated protein 35
A0A4W6CXX6	N-acylglucosamine 2-epimerase (EC 5.1.3.8) (GlcNAc 2-epimerase) (N-acetyl-D-glucosamine 2-epimerase) (Renin-binding protein)
A0A4W6EQR0	Ig-like domain-containing protein
A0A4W6EV01	Dihydrolipoyllysine-residue succinyltransferase component of 2-oxoglutarate dehydrogenase complex, mitochondrial (EC 2.3.1.61) (2-oxoglutarate dehydrogenase complex component E2) (Dihydrolipoamide succinyltransferase component of 2-oxoglutarate dehydrogenase complex) (E2K)
A0A4W6CYS6	Vacuolar protein-sorting-associated protein 25 (ESCRT-II complex subunit VPS25)
A0A4W6CZ50	NACHT, LRR and PYD domains-containing protein 1a
A0A4W6CZP0	Uncharacterized protein
A0AAJ7VKW2	Anamorsin (Cytokine-induced apoptosis inhibitor 1) (Fe-S cluster assembly protein DRE2 homolog)
A0A4W6D046	Thymidine phosphorylase (TP) (EC 2.4.2.4) (TdRPase)
A0A4W6D0U5	Ras-related protein Rab-21
A0A4W6D310	Tubulin alpha chain
A0A4W6D3N3	Proteasome subunit beta
A0AAJ7QDN1	cAMP-dependent protein kinase type I-alpha regulatory subunit
A0A4W6D482	Sorcin
A0AAJ8DNH7	Uncharacterized protein LOC108879369 isoform X1
A0A4W6D6F0	26S proteasome non-ATPase regulatory subunit 6
A0A4W6D7L7	Actin-related protein 3
A0A4W6D8B5	Ubiquitin thioesterase (EC 3.4.19.12)
A0A4W6D8Z0	protein-glutamine gamma-glutamyltransferase (EC 2.3.2.13)
A0A4W6D8F4	Profilin
A0A4W6D8N4	Hydroxylysine kinase (EC 2.7.1.81)
A0A4W6D9F9	Tight junction protein 3
A0A4W6D953	Annexin
A0A4W6DAF4	Small ribosomal subunit protein eS19 (40S ribosomal protein S19)
A0A4W6DAJ1	Apolipoprotein A-IV b, tandem duplicate 2

A0A4W6DAS2	Serine/threonine-protein phosphatase (EC 3.1.3.16)
A0A4W6DAT6	Apolipoprotein C-I
A0A4W6DCH2	Proteasome inhibitor PI31 subunit
A0A4W6DDB2	vesicle-fusing ATPase (EC 3.6.4.6)
A0A4W6DDW7	ATPase H+ transporting accessory protein 1b (V-type proton ATPase subunit S1)
A0A4W6DE16	ADP ribosylation factor 5
A0A4W6DEE0	Adducin 3 (gamma) a
A0A4W6DG29	Coiled-coil domain-containing protein 25
A0A4W6DFA1	Protein Hikeshi
A0A4W6DFG1	Phosphatidylinositol transfer protein beta isoform
A0A4W6DFT7	Glutathione S-transferase A (Glutathione S-transferase rho)
A0A4W6DI67	RNA helicase (EC 3.6.4.13)
A0A4W6DIH5	Trifunctional purine biosynthetic protein adenosine-3 [Includes: Phosphoribosylamine--glycine ligase (EC 6.3.4.13) (Glycinamide ribonucleotide synthetase) (GARS) (Phosphoribosylglycinamide synthetase); Phosphoribosylformylglycinamidine cyclo-ligase (EC 6.3.3.1) (AIR synthase) (AIRS) (Phosphoribosyl-aminoimidazole synthetase); Phosphoribosylglycinamide formyltransferase (EC 2.1.2.2) (5'-phosphoribosylglycinamide transformylase) (GAR transformylase) (GART)]
A0A4W6DIS8	Si:ch211-188p14.4
A0A4W6DJ27	EH domain-containing protein 4 (EH-domain containing 4)
A0A4W6DJ77	Adenylosuccinate synthetase isozyme 2 (AMPSase 2) (AdSS 2) (EC 6.3.4.4) (Adenylosuccinate synthetase, acidic isozyme) (Adenylosuccinate synthetase, liver isozyme) (L-type adenylosuccinate synthetase) (IMP--aspartate ligase 2)
A0A4W6DJJ0	Probable serine carboxypeptidase CPVL
A0A4W6DK36	Alanine--tRNA ligase (EC 6.1.1.7) (Protein lactyltransferase AARS1)
A0A4W6DLA1	Protein-L-isoaspartate O-methyltransferase (EC 2.1.1.77)
A0A4W6DLJ5	RNA helicase (EC 3.6.4.13)
A0A4W6DM92	LIM domain containing preferred translocation partner in lipoma (Lipoma-preferred partner)
A0A4W6DMG4	PITH domain-containing protein 1
A0A4W6DP90	Elongation factor 1-alpha
A0A4W6DPT7	Large ribosomal subunit protein eL21 (60S ribosomal protein L21)
A0A4W6DQT7	Complement component c3a, duplicate 5
A0A4W6DR85	UDP-N-acetylglucosamine pyrophosphorylase 1, like 1 (UDP-N-acetylhexosamine pyrophosphorylase-like protein 1)
A0A4W6DV49	Succinate-semialdehyde dehydrogenase, mitochondrial (EC 1.2.1.24) (NAD(+)-dependent succinic semialdehyde dehydrogenase)
A0A4W6DSA9	Protein phosphatase 1 regulatory subunit 7 (Protein phosphatase 1 regulatory subunit 22)
A0AAJ7LAA4	UTP--glucose-1-phosphate uridylyltransferase (EC 2.7.7.9)
A0A4W6DSJ5	Pentraxin family member
A0A4W6DT94	Actin-related protein 2 (Zgc:101810)
A0AAJ7PXB8	DnaJ homolog subfamily C member 8
A0A4W6DTZ6	Vacuolar protein sorting-associated protein 28 homolog
A0AAJ8B0W9	TIP41-like protein

A0A4W6DWM5	Cytokine receptor-like factor 3
A0A4W6DUA1	Small ribosomal subunit protein uS5 (40S ribosomal protein S2)
A0A4W6DV14	Malate dehydrogenase (EC 1.1.1.37)
A0A4W6DWG5	Adenosine kinase (AK) (EC 2.7.1.20) (Adenosine 5'-phosphotransferase)
A0A4W6E0A4	calcium/calmodulin-dependent protein kinase (EC 2.7.11.17)
A0AAJ7VHB3	AP-2 complex subunit alpha
A0A4W6E3T1	Collagen, type I, alpha 1b
A0A4W6E3V0	Oxidation resistance protein 1
A0A4W6E1N1	Pentraxin family member
A0A4W6E4S9	Large ribosomal subunit protein uL30 (60S ribosomal protein L7)
A0A4W6E244	type I protein arginine methyltransferase (EC 2.1.1.319)
A0A4W6E2A8	Actin-related protein 2
A0AAJ7Q4C3	Calmodulin regulator protein PCP4a isoform X2
A0A4W6E4X1	Complement component 6, duplicate 1 (Complement component C6 isoform X1)
A0A4W6E5M1	Tripartite motif containing 16
A0A4W6E6G8	Uncharacterized protein
A0A4W6E6U7	6-pyruvoyl tetrahydrobiopterin synthase (EC 4.2.3.12)
A0A4W6E7L4	Heterogeneous nuclear ribonucleoprotein A3 (Heteroous nuclear ribonucleoprotein A3)
A0A4W6E8H1	Pentraxin 3 (Pentraxin-related protein PTX3)
A0AAJ7LV11	Secernin-3 isoform X2
A0A4W6EAB5	Vacuolar proton pump subunit B (V-ATPase subunit B) (Vacuolar proton pump subunit B)
A0A4W6ECC4	Large ribosomal subunit protein eL34 (60S ribosomal protein L34)
A0A4W6ECL6	Galectin
A0A4W6ECY8	Ig-like domain-containing protein
A0A4W6EEG1	Rad60/SUMO-like domain-containing protein
A0A4W6EGG2	Myg1 exonuclease (UPF0160 protein MYG1, mitochondrial isoform X2)
A0A4W6EEJ8	SUMO-activating enzyme subunit 2 (EC 2.3.2.-)
A0A4W6EEJ3	Tropomyosin 4
A0A4W6EFX1	START domain-containing protein 10 (PCTP-like protein) (StAR-related lipid transfer protein 10)
A0AAJ7Q484	Serine/threonine-protein phosphatase (EC 3.1.3.16)
A0A4W6EKQ2	Hexokinase-2 (EC 2.7.1.1) (Hexokinase type II)
A0A4W6EJ14	5-hydroxyisourate hydrolase (HIU hydrolase) (HIUHase) (EC 3.5.2.17)
A0A4W6EJJ3	L-xylulose reductase
A0A4W6EP16	Prolyl endopeptidase (EC 3.4.21.-)
A0A4W6ELJ2	Ig-like domain-containing protein
A0AAJ7LPV2	Actin-related protein 2/3 complex subunit 4
A0A4W6ELY7	Acetyl-CoA acetyltransferase 2 (Acetyl-CoA acetyltransferase, cytosolic)
A0A4W6EM27	Translin (Component 3 of promoter of RISC)

A0A4W6EMS0	Dehydrogenase/reductase SDR family member 11 (EC 1.1.1.270) (EC 1.1.1.62) (17-beta-hydroxysteroid dehydrogenase) (3-beta-hydroxysteroid 3-dehydrogenase) (Estradiol 17-beta-dehydrogenase) (Short-chain dehydrogenase/reductase family 24C member 1)
A0A4W6ESR3	Collagen, type I, alpha 2
A0A4W6EPD2	Septin
A0A4W6EPT0	Pyruvate kinase (EC 2.7.1.40)
A0A4W6ENV7	Uncharacterized oxidoreductase Mvan_2161 (Zgc:101765)
A0A4W6ER53	Ig-like domain-containing protein
A0A4W6EPL4	Small ribosomal subunit protein eS1
A0A4W6EVW1	Signal transducer and activator of transcription
A0A4W6EU46	threonine--tRNA ligase (EC 6.1.1.3) (Threonyl-tRNA synthetase)
A0A4W6ERB2	Aspartyl aminopeptidase (EC 3.4.11.21)
A0AAJ7PXZ2	Sulfotransferase (EC 2.8.2.-)
A0A4W6EY81	Interleukin-1 receptor accessory protein
A0A4W6ESX3	Ig-like domain-containing protein
A0A4W6EV96	LPS responsive beige-like anchor protein
A0A4W6EYN3	ADP/ATP translocase (ADP,ATP carrier protein)
A0AAJ7PJ36	Tubulin alpha chain
A0A4W6EX54	Chromosome 11 open reading frame 54 (Ester hydrolase C11orf54 homolog)
A0A4W6EX61	Phosphoserine aminotransferase (EC 2.6.1.52)
A0A4W6F209	KIAA1217
A0A4W6F2K8	Aldo-keto reductase family 7, member A3 (aflatoxin aldehyde reductase)
A0A4W6EZT5	alcohol dehydrogenase (NADP(+)) (EC 1.1.1.2) (S-nitroso-CoA reductase)
A0A4W6F0W2	Four and a half LIM domains protein 1
A0A4W6F5Z3	Argininosuccinate lyase (EC 4.3.2.1) (Arginosuccinase)
A0A4W6F303	Carboxylic ester hydrolase (EC 3.1.1.-)
A0A4W6F494	Poly(rC) binding protein 2
A0A4W6F3K5	Cystathionine beta-synthase (EC 4.2.1.22) (Beta-thionase) (Serine sulfhydrase)
A0A4W6F429	Optineurin
A0AAJ7PH23	Deoxynucleoside triphosphate triphosphohydrolase SAMHD1
A0A4W6F4S0	Protein Churchill
A0AAJ7Q3R6	Septin
A0A4W6F749	Coatomer subunit beta'
A0A4W6F5Z4	Aconitase 1
A0A4W6F6J5	IF rod domain-containing protein
A0A4W6F848	Deoxycytidylate deaminase (EC 3.5.4.12) (dCMP deaminase)
A0A4W6F8D9	Sushi domain-containing protein
A0A4W6FBS4	Homogentisate 1,2-dioxygenase (EC 1.13.11.5) (Homogentisate oxygenase) (Homogentisic acid oxidase) (Homogentisicase)
A0A4W6F8N5	Ig-like domain-containing protein

A0AAJ7PYP8	Leucine-rich repeat flightless-interacting protein 2
A0AAJ7PX08	Delta-aminolevulinic acid dehydratase (EC 4.2.1.24)
A0AAJ7LLB0	GDP-mannose 4,6 dehydratase (EC 4.2.1.47) (GDP-D-mannose dehydratase)
A0A4W6FBS7	ubiquitinyl hydrolase 1 (EC 3.4.19.12)
A0A4W6FE44	Tropomyosin 2 (beta)
A0A4W6FFX3	14 kDa phosphohistidine phosphatase (EC 3.9.1.3) (Phosphohistidine phosphatase 1) (Protein histidine phosphatase)
A0A4W6FDF2	26S proteasome non-ATPase regulatory subunit 4 (26S proteasome regulatory subunit RPN10)
A0A4W6FE18	Isoamyl acetate-hydrolyzing esterase 1 homolog
A0A4W6FE91	Glutathione S-transferase LANCL1 (EC 2.5.1.18) (LanC-like protein 1)
A0A4W6FGC5	Major vault protein
A0A4W6FGA6	Charged multivesicular body protein 2Ba
A0A4W6FJH3	Plexin domain containing 2b
A0AAJ7Q3C8	Charged multivesicular body protein 3
A0A4W6FKS3	Cell division cycle 42, like
A0A4W6FKW8	Coatomer subunit zeta
A0A4W6GAX7	Protein phosphatase 1A (EC 3.1.3.16) (Protein phosphatase 2C isoform alpha)
A0A4W6FLP1	Platelet-activating factor acetylhydrolase IB subunit alpha2 (EC 3.1.1.47) (PAF acetylhydrolase 30 kDa subunit) (PAF-AH subunit beta)
A0A4W6FLQ0	Uncharacterized protein wu:fc46h12
A0A4W6FM20	Ig-like domain-containing protein
A0A4W6FNH1	Complement component 8, gamma polypeptide (Complement component C8 gamma chain)
A0A4W6FNJ7	Ig-like domain-containing protein
A0A4W6FNP8	Ig-like domain-containing protein
A0A4W6FNU4	Ependymin
A0A4W6FP12	N-acetylmuramoyl-L-alanine amidase (Peptidoglycan recognition protein 2)
A0AAJ7QJ59	Phosphatidylinositol 5-phosphate 4-kinase type-2 alpha (EC 2.7.1.149) (1-phosphatidylinositol 5-phosphate 4-kinase 2-alpha) (Diphosphoinositide kinase 2-alpha) (Phosphatidylinositol 5-phosphate 4-kinase type II alpha) (PtdIns(5)P-4-kinase isoform 2-alpha)
A0A4W6FRZ2	4-hydroxyphenylpyruvate dioxygenase
A0A4W6FRI0	Thiamine pyrophosphokinase 1
A0A4W6FSH7	Eukaryotic translation initiation factor 3 subunit K (eIF3k) (Eukaryotic translation initiation factor 3 subunit 12) (eIF-3 p25)
A0A4W6FSV4	Uncharacterized protein
A0A4W6FTC3	Apolipoprotein M
A0A4W6FTU8	Uncharacterized protein
A0A4W6FUB0	Perilipin
A0AAJ7L7P6	Calpain-3 isoform X2
A0AAJ7PXH5	Lactadherin isoform X4
A0A4W6FV78	S100 calcium binding protein W
A0A4W6FW75	Small ribosomal subunit protein uS12 (40S ribosomal protein S23)
A0A4W6FWY2	Protein farnesyltransferase subunit beta (FTase-beta) (EC 2.5.1.58)

A0A4W6G0C9	Synapse-associated protein 1 (BSD domain-containing signal transducer and Akt interactor protein)
A0A4W6G1L8	15-oxoprostaglandin 13-reductase (EC 1.3.1.48)
A0A4W6G3X5	N-myc-interactor
A0A4W6G4J2	small monomeric GTPase (EC 3.6.5.2)
A0A4W6G571	Deoxyribonuclease
A0AAJ8B4D0	Aspartyl/asparaginyl beta-hydroxylase isoform X5
A0A4W6G5V5	NIF3-like protein 1
A0AAJ7L8B5	Carboxypeptidase B2
A0A4W6G6B8	PDZ and LIM domain protein 2
A0A4W6G6Z2	Cofilin-2
A0AAJ7PHK5	Beta-microseminoprotein-like
A0A4W6G985	Caspase-9 (EC 3.4.22.62) (Apoptotic protease Mch-6) (Apoptotic protease-activating factor 3) (ICE-like apoptotic protease 6)
A0A4W6G7F7	SUEL-type lectin domain-containing protein
A0AAJ8B1I7	Cephalotoxin-like protein
A0A4W6G9P7	Versican core protein (Chondroitin sulfate proteoglycan core protein 2) (Large fibroblast proteoglycan) (PG-M)
A0A4W6G925	GDNF family receptor alpha-2 (GDNF receptor alpha-2)
A0A4W6G9L5	Hyaluronan and proteoglycan link protein 1
A0A4W6G9R8	Methyltransferase DDB_G0268948 (Zgc:162396)
A0AAJ7LLA3	Transcobalamin-2
A0A4W6GBG9	Phosphoinositide-3-kinase-interacting protein 1
A0AAJ7LL54	PG-M
A0AAJ7LPZ7	Uncharacterized protein LOC108881594
A0AAJ7LQ82	Uncharacterized protein LOC108880687
A0AAJ7LQL2	LOW QUALITY PROTEIN: putative aminopeptidase W07G4.4
A0AAJ7LUV6	Glutathione peroxidase
A0AAJ7PKD0	Large ribosomal subunit protein eL6 (60S ribosomal protein L6)
A0AAJ8DXI2	Septin
A0AAJ7Q216	Insulin-like growth factor-binding protein 7
A0AAJ7Q8V7	histidine--tRNA ligase (EC 6.1.1.21)
A0AAJ7QAR9	Programmed cell death protein 4
A0AAJ7QB06	Large ribosomal subunit protein uL6 (60S ribosomal protein L9)
A0AAJ7QDD8	Lymphocyte antigen 6G
A0AAJ7QDG6	Calpain-2 catalytic subunit
A0AAJ7QFZ2	Myristoylated alanine-rich C-kinase substrate
A0AAJ8DXI8	Protein POF1B isoform X3
A0AAJ7QLC0	A-kinase anchor protein 12
A0AAJ7QN90	LOW QUALITY PROTEIN: hemoglobin subunit alpha-A-like
A0AAJ7V3C6	Zinc finger protein ZPR1 (Zinc finger protein 259)

A0AAJ7V9I8	E1 ubiquitin-activating enzyme (EC 6.2.1.45) (Ubiquitin-activating enzyme E1)
A0AAJ7VGE2	LOW QUALITY PROTEIN: coiled-coil domain-containing protein 6
A0AAJ7VGQ9	Bifunctional glutamate/proline--tRNA ligase (EC 6.1.1.15) (EC 6.1.1.17) (Bifunctional aminoacyl-tRNA synthetase)
A0AAJ7VKD2	5'-nucleotidase (EC 3.1.3.5)
A0AAJ8AXG1	Epidermal growth factor receptor kinase substrate 8-like protein 1
A0AAJ8B987	Versican core protein (Chondroitin sulfate proteoglycan core protein 2) (Large fibroblast proteoglycan) (PG-M)
A0AAJ8BCM0	LOW QUALITY PROTEIN: collagen alpha-5(IV) chain-like
A0AAJ8BH02	Mucin-5AC
A0AAJ8BKD0	LOW QUALITY PROTEIN: neuroblast differentiation-associated protein AHNAK
A0AAJ8BM86	Uncharacterized protein LOC108897942
A0AAJ8DLN3	ADP-sugar pyrophosphatase (EC 2.7.7.96) (EC 3.6.1.13) (EC 3.6.1.58) (8-oxo-dGDP phosphatase) (Nuclear ATP-synthesis protein NUDIX5) (Nucleoside diphosphate-linked moiety X motif 5)
A0AAJ8DM39	LOW QUALITY PROTEIN: uncharacterized protein LOC108893007
A0AAJ8DQS6	LOW QUALITY PROTEIN: apolipoprotein B-100-like
A0AAJ8DS54	Uncharacterized protein LOC127138918
A0AAJ8DVL2	Neuroblast differentiation-associated protein AHNAK
A0AAJ8DWW0	Uncharacterized protein LOC108897954

Table S5: Differentially abundant protein groups in the skin mucus of male and female barramundi determined by LFQ-based analysis with adjusted p-values (FDR < 0.05).

UniProt Accession	Putative protein annotations	logFC	Adj. p-val
**DAPs with increased abundance**			
A0A4W6DWS9	14-3-3 domain-containing protein	2.97854678	0.00384851
A0A4W6EZ60	14-3-3 domain-containing protein	1.1871287	0.02779814
A0A4W6FFC4	14-3-3 protein beta/alpha-1 (Tyrosine 3-monooxygenase/tryptophan 5-monooxygenase activation protein, theta polypeptide b)	1.41914749	0.02048794
A0A4W6EV82	14-3-3 protein beta/alpha-A (Tyrosine 3-monooxygenase/tryptophan 5-monooxygenase activation protein beta)	1.46558317	0.02301136
A0A4W6BJP2	14-3-3 protein epsilon	1.69094467	0.01952733
A0A4W6DLL8	15-hydroxyprostaglandin dehydrogenase [NAD(+)] (EC 1.1.1.141) (EC 1.1.1.232) (Eicosanoid/docosanoid dehydrogenase [NAD(+)]) (Prostaglandin dehydrogenase 1)	3.67929331	0.00122848
A0A4W6CU94	2',3'-cyclic-nucleotide 3'-phosphodiesterase (EC 3.1.4.37)	1.01395734	0.03897773
A0A4W6FTR5	26S proteasome non-ATPase regulatory subunit 12 (26S proteasome regulatory subunit RPN5)	1.1969525	0.04306048
A0A4W6CWS7	26S proteasome non-ATPase regulatory subunit 14 (26S Proteasome regulatory subunit RPN11)	1.20175648	0.03126007
A0A4W6EG21	5'-nucleotidase, cytosolic IB b (Cytosolic 5'-nucleotidase 1A)	1.15698179	0.03864954

A0AAJ7QMJ2	6-phosphogluconolactonase (6PGL) (EC 3.1.1.31)	1.08297157	0.03897773
A0AAJ7QA49	Acireductone dioxygenase (Acireductone dioxygenase (Fe(2+)-requiring)) (Acireductone dioxygenase (Ni(2+)-requiring)) (Membrane-type 1 matrix metalloproteinase cytoplasmic tail-binding protein 1) (ARD) (ARD') (Fe-ARD) (MTCBP-1) (Ni-ARD) (EC 1.13.11.53, EC 1.13.11.54)	1.02105395	0.03656496
A0A4W6DT31	Actin-related protein 2	1.97483762	0.01952733
A0A4W6DEJ2	Actin-related protein 2/3 complex subunit 3	1.06036123	0.03125017
A0A4W6F614	Actin-related protein 2/3 complex subunit 4	1.5207208	0.0279256
A0A4W6E7B1	Actin-related protein 3	1.57877668	0.01984295
A0AAJ7LBU9	Actin, cytoplasmic 1	2.69668833	0.01693908
A0A4W6DCW0	Actin, cytoplasmic 2	1.34858704	0.02904856
A0A4W6FX29	Actinin alpha 4 (Alpha-actinin-4 isoform X1)	1.51871936	0.02048794
A0AAJ7LW96	Adenosylhomocysteinase (EC 3.13.2.1)	1.12108485	0.04293502
A0A4W6FA46	Alcohol dehydrogenase 5-like (Alcohol dehydrogenase class-3)	1.35287793	0.03080221
A0A4W6CCH8	Alpha-1-antitrypsin homolog	2.23767535	0.01984295
A0A4W6CEC6	Alpha-1-antitrypsin homolog	1.81592433	0.02609892
A0A4W6FP82	Alpha-1,4 glucan phosphorylase (EC 2.4.1.1)	2.11743609	0.02048794
A0A4W6FPN5	Alpha-2-HS-glycoprotein 2	2.15647507	0.01984295
A0AAJ7LPC5	Alpha-aminoadipic semialdehyde dehydrogenase (EC 1.2.1.3) (EC 1.2.1.31) (EC 1.2.1.8) (Aldehyde dehydrogenase family 7 member A1) (Antiquitin-1) (Betaine aldehyde dehydrogenase) (Delta1-piperideine-6-carboxylate dehydrogenase)	1.12924576	0.03080221
A0A4W6C9M7	Alpha-galactosidase (EC 3.2.1.-)	1.62272708	0.02727905
A0AAJ7LV51	Aminopeptidase (EC 3.4.11.-)	1.79007467	0.01952733
A0A4W6FYC7	Aminopeptidase like 1	1.61462402	0.03369162
A0A4W6FWP4	Antithrombin-III (Serpin C1)	1.87510363	0.02048794
A8D3K0	Apolipoprotein	3.22212029	0.01952733
A0A4W6DST3	Apolipoprotein A-I	2.16487694	0.02371699
A0A4W6FSI6	Apolipoprotein M	2.74045054	0.0355152
A0A4W6F648	Apolipoprotein M	1.29857	0.03919744
A0A4W6F3W1	Arp2/3 complex 34 kDa subunit	1.35928027	0.0348864
A0A4W6CUH4	ATP-citrate synthase (EC 2.3.3.8) (ATP-citrate (pro-S-)-lyase) (Citrate cleavage enzyme)	1.43822289	0.02021779
A0A4W6FSV5	Beta-2-glycoprotein 1 (Apolipoprotein H) (Beta-2-glycoprotein I)	1.82081922	0.02874335
A0A4W6EYE6	Biotinidase (EC 3.5.1.12)	2.29546102	0.02048794
A0A4W6EVI7	Biotinidase (EC 3.5.1.12)	1.79229101	0.02048794
A0AAJ7LJ19	Biotinidase (EC 3.5.1.12)	1.75782267	0.02301136
A0A4W6E9H2	BTB/POZ domain-containing protein	1.64363257	0.02301136
A0A4W6G058	C-type lectin domain-containing protein	2.19713847	0.0397402
A0A4W6F4X9	C1q domain-containing protein	1.7310009	0.04984352
A0AAJ7VEW7	Cadherin-1	2.03148524	0.01693908

A0A4W6DKM4	Calcium/calmodulin dependent protein kinase I	2.0086937	0.02070943
A0AAJ7LHZ1	calpain-2 (EC 3.4.22.53)	2.4649601	0.04198832
A0A4W6CUY1	Calpastatin (Calpain inhibitor)	1.32403692	0.02727905
A0AAJ7PFJ9	cAMP-regulated phosphoprotein 19a	1.60186386	0.01984295
A0A4W6FGV7	Carboxypeptidase (EC 3.4.16.-)	1.34831556	0.02100784
A0A4W6EJA6	Caspase-3 (EC 3.4.22.56)	1.08030446	0.04198832
A0A4W6DFE4	Catechol O-methyltransferase domain-containing protein 1 (Catechol-O-methyltransferase domain containing 1)	1.92742856	0.02371699
A0A4W6G0W8	Cathepsin B (EC 3.4.22.1)	1.21420797	0.02371699
A0A4W6EIR0	CD44 antigen (GP90 lymphocyte homing/adhesion receptor) (HUTCH-I) (Hermes antigen) (Hyaluronate receptor) (Phagocytic glycoprotein 1) (Phagocytic glycoprotein I)	1.09011078	0.03125017
A0A4W6FJQ6	Charged multivesicular body protein 1B	1.1491375	0.04239699
A0A4W6CDI7	Chloride intracellular channel protein 2 (Glutaredoxin-like oxidoreductase CLIC2) (Glutaredoxin-like peroxidase CLIC2)	1.32335218	0.04669546
A0AAJ7LSP9	Chymotrypsinogen A	1.46617063	0.03468276
A0A4W6FRY8	Coatomer subunit epsilon	1.33079084	0.02371699
A0AAJ8B6R0	Complement C3 isoform X1	2.72289149	0.04167792
A0A4W6EIU1	Complement component c3b, tandem duplicate 2	2.26018906	0.02769759
A0A4W6EED3	Complement component c3b, tandem duplicate 2	2.35789617	0.03080221
A0AAJ7LW10	Complement factor H isoform X2	1.45550156	0.04970845
A0A4W6FRD2	CXXC motif containing zinc binding protein	1.06535657	0.02301136
A0A4W6DL93	Cystatin-B (Stefin-B)	1.05784925	0.02371699
A0A4W6E496	Cystatin-B (Stefin-B)	1.47979609	0.04669546
A0A4W6EMW7	Cysteine and glycine-rich protein 1 (Cysteine-rich protein 1)	1.67929268	0.02048794
A0A4W6CHE3	Cysteine--tRNA ligase, cytoplasmic (EC 6.1.1.16) (Cysteinyl-tRNA synthetase)	1.44524066	0.02371699
A0AAJ7PIS3	Cytosolic non-specific dipeptidase (EC 3.4.13.18) (CNDP dipeptidase 2) (Threonyl dipeptidase)	1.42069181	0.03080221
A0A4W6FMB4	D-3-phosphoglycerate dehydrogenase (EC 1.1.1.95)	1.68839137	0.03308591
A0A4W6DM19	Dehydrogenase/reductase SDR family member 11 (EC 1.1.1.270) (EC 1.1.1.62) (17-beta-hydroxysteroid dehydrogenase) (3-beta-hydroxysteroid 3-dehydrogenase) (Estradiol 17-beta-dehydrogenase) (Short-chain dehydrogenase/reductase family 24C member 1)	1.30437469	0.01952733
A0A4W6DNW2	Dihydrolipoyl dehydrogenase (EC 1.8.1.4)	2.85435994	0.01952733
A0A4W6DIT2	DnaJ heat shock protein family (Hsp40) member B1b (DnaJ homolog subfamily B member 1b)	1.10516485	0.02301136
A0A4W6EU56	E1 ubiquitin-activating enzyme (EC 6.2.1.45) (Ubiquitin-activating enzyme E1)	1.04500135	0.04683375
A0A4W6E2Z9	E2 ubiquitin-conjugating enzyme (EC 2.3.2.23)	1.02637355	0.02727905
A0A4W6EIK0	E2 ubiquitin-conjugating enzyme (EC 2.3.2.23)	1.0306956	0.03308591
A0A4W6BXR7	EF-hand domain family, member D1	1.55455907	0.01984295
A0A4W6CXK9	EF-hand domain family, member D2 (EF-hand domain-containing protein D2)	1.02991486	0.03125017
A0A4W6F112	EGF-like domain-containing protein	2.46533012	0.03241339

A0AAJ7QFF9	Elongation factor 1-alpha	1.12581062	0.02070943
A0A4W6E4G8	Elongin-C (Elongin 15 kDa subunit) (RNA polymerase II transcription factor SIII subunit C) (SIII p15) (Transcription elongation factor B polypeptide 1)	1.29709625	0.02727905
A0A4W6EYN2	Endoplasmin (Heat shock protein 90 kDa beta member 1)	1.35006587	0.04670082
A0AAJ7QF13	Envoplakin	1.05951691	0.03125017
A0A4W6DED5	Eukaryotic translation initiation factor 1	1.41585223	0.01984295
A0A4W6F7K4	Fetuin B	1.53268878	0.02371699
A0A4W6F7K9	Fetuin-B	1.72938792	0.0356189
A0AAJ7LD03	Fibrinogen alpha chain	1.86064529	0.04217829
A0AAJ7QGK7	Filamin-A isoform X3	1.38245265	0.03125017
A0A4W6FI10	Fructose-1,6-bisphosphatase 1 (EC 3.1.3.11) (D-fructose-1,6-bisphosphate 1-phosphohydrolase 1) (Liver FBPase)	1.28379122	0.03897773
A0A4W6G6D1	Fructose-bisphosphate aldolase (EC 4.1.2.13)	1.12547048	0.02904856
A0AAJ7PTR7	Galactose-specific lectin nattectin	1.31482442	0.04009024
A0A4W6FIV4	Glia maturation factor	1.14740181	0.0370674
A0A4W6BXL4	Glutaredoxin 3	1.80336316	0.01952733
A0A4W6DGU5	Glutathione S-transferase A (Glutathione S-transferase rho)	1.22452037	0.04863538
A0A4W6CJ78	glutathione transferase (EC 2.5.1.18) (GST class-mu)	1.2276001	0.03125017
A0A4W6EDU9	Glyceraldehyde-3-phosphate dehydrogenase (EC 1.2.1.12)	2.3508021	0.01952733
A0A4W6DHL2	Glycine N-methyltransferase (EC 2.1.1.20)	1.08325259	0.03199305
A0A4W6EWH0	Haptoglobin	3.12466558	0.02048794
A0A4W6CXE8	Hcy-binding domain-containing protein	1.99991989	0.01984295
A0AAJ7QK86	Hemopexin	2.2362086	0.01952733
A0AAJ8BMK0	hexokinase (EC 2.7.1.1)	1.43713951	0.03080221
A0AAJ7PLH2	High mobility group nucleosomal binding domain 7	1.21850332	0.02727905
A0AAJ7PIL1	Hydroxyacylglutathione hydrolase, mitochondrial (EC 3.1.2.6) (Glyoxalase II)	1.24608167	0.03086328
A0AAJ8DT66	Ig heavy chain Mem5	2.42121633	0.03093048
A0A4W6F9K7	Ig-like domain-containing protein	2.76477877	0.01693908
A0A4W6EMK6	Ig-like domain-containing protein	2.29568418	0.02371699
A0A4W6CKL7	Ig-like domain-containing protein	1.62540754	0.02727905
A0A4W6EPU4	Ig-like domain-containing protein	2.53306262	0.03125017
A0A4W6FLD5	Ig-like domain-containing protein	1.9695975	0.03316696
A0A4W6FNL2	Ig-like domain-containing protein	2.35698732	0.03897773
A0A4W6EM85	Ig-like domain-containing protein	2.60921478	0.03686237
A0A4W6EQC4	Ig-like domain-containing protein	2.21609497	0.03897773
A0A4W6EUY1	Inosine-uridine preferring nucleoside hydrolase	1.66484578	0.03125017
A0A4W6EYC6	Integrin alpha-6 isoform X2 (Integrin, alpha 6b)	1.30399386	0.03897773
A0A4W6EX30	Inter-alpha-trypsin inhibitor heavy chain 3b, tandem duplicate 1	2.208402	0.02070943

A0AAJ7V4B6	Inter-alpha-trypsin inhibitor heavy chain H3	3.58746783	0.03897773
A0A4W6D1V5	Isocitrate dehydrogenase [NADP] (EC 1.1.1.42)	1.04280535	0.04970845
A0A4W6EVC2	IST1 homolog (Charged multivesicular body protein 8)	1.00639216	0.03125017
A0AAJ7LXX1	Keratin, type I cytoskeletal 18	3.28539022	0.03919744
A0AAJ7LNV5	Kininogen-1 isoform X2	1.86634	0.02551768
A0AAJ8B9V1	Leucine-rich alpha-2-glycoprotein	1.82902336	0.04027554
A0AAJ7PMZ2	Leucine-rich repeat-containing protein DDB_G0290503 isoform X1	2.56289927	0.03241339
A0AAJ8AZQ0	Leukocyte elastase inhibitor (Serpin B1) (Serpin B6)	1.06413905	0.02769759
A0AAJ7Q5F1	Lipocalin-like isoform X2	1.51859315	0.03308591
A0AAJ7VIB2	LOW QUALITY PROTEIN: actin, cytoplasmic 1	1.64835103	0.01952733
A0AAJ7VDX8	LOW QUALITY PROTEIN: alpha-2-macroglobulin-like	2.33020528	0.02021779
A0AAJ8DJW2	LOW QUALITY PROTEIN: alpha-2-macroglobulin-like	2.18855158	0.02049118
A0AAJ8B9I2	LOW QUALITY PROTEIN: apolipoprotein Bb, tandem duplicate 1	2.83699608	0.04669546
A0AAJ7QL81	LOW QUALITY PROTEIN: arfaptin-1	1.33428955	0.02371699
A0AAJ8B8Z7	LOW QUALITY PROTEIN: complement factor I-like	1.87566884	0.03897773
A0AAJ7LCV8	LOW QUALITY PROTEIN: junction plakoglobin a	1.48438136	0.02070943
A0AAJ8DT00	LOW QUALITY PROTEIN: uncharacterized protein LOC108880742	1.92131933	0.03897773
A0A4W6E3V7	Lumican	2.64001465	0.01952733
A0A4W6G7E8	Malate dehydrogenase (EC 1.1.1.37)	1.24216461	0.02952001
A0AAJ7LDE8	mannose-1-phosphate guanylyltransferase (EC 2.7.7.13)	1.1447099	0.02727905
A0A4W6E222	Matrix metallopeptidase 30	1.63370005	0.04306048
A0AAJ7PEF6	Myosin-9 (Myosin heavy chain 9) (Myosin heavy chain, non-muscle IIa) (Non-muscle myosin heavy chain IIa)	2.30071322	0.04970845
A0A4W6D3J8	N-acetylneuraminate-9-phosphate synthase (EC 2.5.1.57) (Sialic acid synthase)	1.25639407	0.02301136
A0A4W6C8L4	N-acylneuraminate cytidylyltransferase (EC 2.7.7.43)	2.83025996	0.00933399
A0AAJ7LQB2	N-alpha-acetyltransferase 50 (EC 2.3.1.258) (N-epsilon-acetyltransferase 50) (NatE catalytic subunit)	1.67855008	0.02048794
A0A4W6E467	NAD(P)H-hydrate epimerase (EC 5.1.99.6) (Apolipoprotein A-I-binding protein) (AI-BP) (NAD(P)HX epimerase)	2.10774612	0.01693908
A0A4W6FNI6	Neurofilament heavy polypeptide	1.20808729	0.03241339
A0A4W6FH89	NHL repeat-containing protein 3	1.07787005	0.0414368
A0A4W6CC48	non-specific serine/threonine protein kinase (EC 2.7.11.1)	1.08861415	0.03080221
A0A4W6EII3	Nucleoside diphosphate kinase (EC 2.7.4.6)	1.34024556	0.02301136
A0A4W6D811	Papilin	1.83629767	0.02301136
A0A4W6DDB9	Pdgfa associated protein 1b	1.45320892	0.04275434
A0A4W6D1T0	PDZ and LIM domain 5b	1.02903112	0.03897773
A0A4W6E288	peptidylprolyl isomerase (EC 5.2.1.8)	1.06446266	0.02371699
A0A4W6E239	peptidylprolyl isomerase (EC 5.2.1.8)	1.02358564	0.0374351
A0A4W6F480	Peroxiredoxin-1 (EC 1.11.1.24)	1.09578578	0.02464317

A0AAJ7PVB0	Phosphatidylinositol transfer protein alpha isoform	1.23445892	0.02727905
A0A4W6C6V9	Pigment epithelium-derived factor	1.92415937	0.03897773
A0AAJ7LHL5	Plakophilin-1 isoform X2	2.16407267	0.01952733
A0AAJ7QAF2	Plastin-2	1.27728462	0.03125017
A0AAJ7Q675	Plastin-3 (T-plastin)	1.73092143	0.02727905
A0AAJ8AZ56	Pleckstrin homology domain-containing family A member 1 isoform X2 (Pleckstrin homology domain-containing family A member 1 isoform X3) (Pleckstrin homology domain-containing family A member 1 isoform X4) (Pleckstrin homology domain-containing family A member 1 isoform X5)	1.20719528	0.04198832
A0A4W6CDL0	Prefoldin subunit 3	1.02722232	0.03292424
A0A4W6D5A2	Pro-interleukin-16 [Cleaved into: Interleukin-16 (IL-16) (Lymphocyte chemoattractant factor) (LCF)]	1.38982073	0.01952733
A0A4W6EMQ5	Proliferation-associated 2G4, a	1.36745199	0.01952733
A0AAJ7QNI8	Prolyl endopeptidase (EC 3.4.21.-)	1.88441022	0.01693908
A0A4W6ELV3	Proteasome 26S subunit, non-ATPase 11	1.41320419	0.02301136
A0A4W6G5R0	Proteasome activator complex subunit 2 (Proteasome activator 28 subunit beta)	1.35001437	0.03308591
A0A4W6F3X8	proteasome endopeptidase complex (EC 3.4.25.1)	1.08401489	0.03316696
A0A4W6CCD4	Proteasome subunit alpha type	1.01456451	0.03292424
A0A4W6F858	Proteasome subunit beta	1.10799535	0.04321448
A0AAJ7PI43	Protein AMBP	1.67429415	0.0348864
A0A4W6CJK3	Protein MEMO1 (Mediator of ErbB2-driven cell motility 1)	1.08473396	0.03600729
A0A4W6FTF0	Protein S100 (S100 calcium-binding protein)	2.12085915	0.01693908
A0A4W6FDQ8	Protein S100 (S100 calcium-binding protein)	1.94852924	0.03369162
A0A4W6C042	Protein Z, vitamin K-dependent plasma glycoprotein a	1.32166481	0.01984295
A0A4W6DU43	Prothrombin (EC 3.4.21.5) (Coagulation factor II)	1.89228948	0.01984295
A0A4W6EP73	Pyruvate kinase (EC 2.7.1.40)	1.47745959	0.01984295
A0A4W6DAX0	Rho GDP-dissociation inhibitor 1 (Rho-GDI alpha)	1.04551697	0.03080221
A0A4W6C5D3	Ribosyldihydronicotinamide dehydrogenase [quinone] (EC 1.10.5.1) (NRH dehydrogenase [quinone] 2) (NRH:quinone oxidoreductase 2) (Quinone reductase 2)	1.30854607	0.02070943
A0A4W6FA37	S-(hydroxymethyl)glutathione dehydrogenase (EC 1.1.1.284)	1.68896866	0.04167792
A0A4W6DSS5	S-phase kinase-associated protein 1	1.73088582	0.01693908
A0A4W6CYI8	Scinderin like a	1.64779981	0.01984295
A0AAJ7PMH9	Semaphorin-1A	1.09261703	0.03417579
A0A4W6DCP4	Serine--tRNA ligase, cytoplasmic (EC 6.1.1.11) (Seryl-tRNA synthetase)	1.65840658	0.04669546
A0A4W6DHK6	Serine/threonine-protein phosphatase (EC 3.1.3.16)	1.44607862	0.02048794
A0A4W6DWW9	Serine/threonine-protein phosphatase (EC 3.1.3.16)	1.58417702	0.02070943
A0A4W6G4L1	Serine/threonine-protein phosphatase 2A 55 kDa regulatory subunit B	1.59593074	0.0279256
A0AAJ7PXA5	Serine/threonine-protein phosphatase CPPED1 (EC 3.1.3.16) (Calcineurin-like phosphoesterase domain-containing protein 1)	1.30128415	0.04691345
A0A4W6C6U3	Serpin domain-containing protein	2.32078997	0.02070943

A0AAJ7V9S5	Serpin peptidase inhibitor, clade A (Alpha-1 antiproteinase, antitrypsin), member 10a	2.41351382	0.01693908
A0A4W6CUF5	Sex hormone-binding globulin	2.29757404	0.01984295
A0AAJ7Q8Y5	SH3 domain-binding glutamic acid-rich-like protein 3	1.04048665	0.04863624
A0A4W6BKI9	Si:ch1073-126c3.2	2.62071673	0.02301136
A0A4W6DYB6	Si:ch211-76l23.4	2.49278641	0.02048794
A0A4W6CC08	Si:ch73-22o12.1	1.57039165	0.03086328
A0A4W6E2B4	Sialidase-1 (EC 3.2.1.18) (Lysosomal sialidase) (N-acetyl-alpha-neuraminidase 1)	1.3001407	0.03593695
A0A4W6EWN6	Small ribosomal subunit protein uS2 (37 kDa laminin receptor precursor) (37LRP) (37/67 kDa laminin receptor) (LRP/LR) (67 kDa laminin receptor) (67LR) (Laminin receptor 1) (LamR) (Laminin-binding protein precursor p40) (LBP/p40)	1.78550275	0.0289823
A0AAJ8DR46	Soluble scavenger receptor cysteine-rich domain-containing protein SSC5D	1.79907227	0.03919744
A0A4W6C0P9	Sorting nexin	1.3206679	0.03308591
A0A4W6CF23	Spermidine synthase (EC 2.5.1.16) (Putrescine aminopropyltransferase)	1.22128995	0.03130095
A0AAJ7PIE8	Staphylococcal nuclease domain-containing protein (EC 3.1.31.1)	1.00637372	0.02727905
A0AAJ7LQF9	Sugar phosphate phosphatase (EC 2.1.1.-) (EC 3.1.3.-)	1.43102455	0.0285283
A0A4W6D6V8	Sulfurtransferase	1.0179081	0.03308591
A0A4W6C1S3	Superoxide dismutase [Cu-Zn] (EC 1.15.1.1)	1.92032337	0.03007503
A0A4W6FIR8	Synaptic vesicle membrane protein VAT-1 homolog (Vesicle amine transport 1)	1.193367	0.02727905
A0A4W6DAG4	T-complex protein 1 subunit theta (CCT-theta)	1.59313965	0.01952733
A0A4W6DRS5	THAP domain containing 4 (THAP domain-containing protein 4)	1.36361059	0.01952733
A0A4W6FHT9	thioredoxin-disulfide reductase (EC 1.8.1.9)	1.88473701	0.01693908
A0A4W6G2H2	Tpd52 like 2b	1.1059316	0.02070943
A0AAJ7Q641	Transgelin	1.77827835	0.02371699
A0A4W6DQ20	TRPM8 channel-associated factor homolog	2.35213025	0.03919744
A0A4W6C9V6	trypsin (EC 3.4.21.4)	1.84297053	0.02371699
A0A4W6DI18	trypsin (EC 3.4.21.4)	1.58511925	0.03080221
A0A4W6DVM2	Tryptase-2	3.06838671	0.03600729
A0A4W6EWG5	Tubulin alpha chain	1.28693136	0.03308591
A0A4W6DD42	Ubiquitin carboxyl-terminal hydrolase (EC 3.4.19.12)	1.62098503	0.02048794
A0A4W6CPX7	Ubiquitin-like protein NEDD8 (Neddylin)	1.17619642	0.02371699
A0A4W6CQ70	Uncharacterized oxidoreductase YjmC isoform X2	4.27862231	0.01952733
A0A4W6EP66	Uncharacterized protein	1.44754696	0.02371699
A0A4W6DQ31	Uncharacterized protein	1.67448934	0.03086328
A0A4W6FTB3	Uncharacterized protein	1.60303624	0.04332274
A0AAJ7PEF2	Uncharacterized protein LOC108875889	2.68034299	0.02048794
A0A4W6G889	Uncharacterized protein LOC108878177	2.16915989	0.01984295
A0AAJ7Q3K1	Uncharacterized protein LOC108892627	2.1999321	0.02301136
A0A4W6F654	Uncharacterized protein LOC108892780	1.89992015	0.02070943

A0AAJ7QMK5	Uncharacterized protein LOC108902583	2.86182276	0.01984295
A0AAJ7LED4	UPF0462 protein C4orf33 homolog isoform X2	1.09104156	0.03080221
A0AAJ7PSW0	V-type proton ATPase subunit C	1.54040464	0.02301136
A0A4W6BYA4	Vesicle amine transport 1	1.01308695	0.04670082
A0A4W6C222	Vinculin (Metavinculin)	1.40324847	0.03366785
A0AAJ7VHR3	von Willebrand factor A domain-containing protein 5A	1.34172185	0.02371699
A0A4W6G1R9	VPS26, retromer complex component B (Vacuolar protein sorting-associated protein 26B-like)	1.96891721	0.01984295
A0AAJ7LWU9	WAS/WASL-interacting protein family member 2	1.14590518	0.04167792
A0A4W6D4A0	WD repeat domain 1	2.13070043	0.03125017
**DAPs with decreased abundance**			
A0A4W6CLJ5	Charged multivesicular body protein 5 (Chromatin-modifying protein 5)	-1.1528053	0.03125017
A0A4W6CWU2	Chromosome 2 open reading frame 76 (UPF0538 protein C2orf76 homolog)	-1.6882032	0.02048794
A0AAJ7LZL5	Dermatopontin-like	-2.2530575	0.04669546
A0AAJ7QDZ5	High mobility group nucleosome-binding domain-containing protein 3	-2.7129148	0.0106877
A0A4W6C9V7	Homer protein homolog 2 isoform X3 (Homer scaffold protein 2)	-1.2326298	0.03455806
A0A4W6FME3	Ig-like domain-containing protein	-1.7936398	0.03365385
A0A4W6EY32	Interleukin 4/13A	-1.8952443	0.02301136
A0AAJ7PXZ7	Keratin, type II cytoskeletal 8 isoform X2	-2.3017454	0.04669546
A0AAJ8AZV1	Leukocyte elastase inhibitor (Serpin B1) (Serpin B6)	-2.6653862	0.01693908
A0AAJ8DQ34	LOW QUALITY PROTEIN: keratin 94	-1.5559254	0.03365385
A0AAJ7QCN1	LOW QUALITY PROTEIN: lamina-associated polypeptide 2, isoforms beta/delta/epsilon/gamma-like	-1.9082731	0.02371699
A0A4W6E6U5	Microfibril associated protein 4 (Microfibril-associated glycoprotein 4)	-3.3071963	0.01952733
A0AAJ8BIN7	NAD(P)(+)--arginine ADP-ribosyltransferase (EC 2.4.2.31) (Mono(ADP-ribosyl)transferase)	-3.7541498	0.03897773
A0A4W6FQ72	Pyruvate kinase (EC 2.7.1.40)	-1.6395359	0.02049118
A0AAJ7PH03	Trypsin-4	-2.1294994	0.03919744
A0A4W6DP83	Zona pellucida sperm-binding protein 3	-3.3257742	0.0306392
